# Scalable single-cell profiling of chromatin modifications with sciCUT&Tag

**DOI:** 10.1038/s41596-023-00905-9

**Published:** 2023-11-07

**Authors:** Derek H. Janssens, Jacob E. Greene, Steven J. Wu, Christine A. Codomo, Sam S. Minot, Scott N. Furlan, Kami Ahmad, Steven Henikoff

**Affiliations:** 1. Basic Sciences Division, Fred Hutchinson Cancer Center, Seattle, WA, USA.; 2. Molecular Medicine and Mechanisms of Disease (M3D) PhD Program, University of Washington, Seattle, WA, USA.; 3. Molecular Engineering & Sciences Institute, University of Washington, Seattle, WA, USA; 4. Data Core, Fred Hutchinson Cancer Center, Seattle, WA, USA.; 5. Clinical Research Division, Fred Hutchinson Cancer Center, Seattle, WA, USA.; 6. Department of Pediatrics, University of Washington, Seattle, WA, USA.; 7. Brotman-Baty Institute for Precision Medicine, University of Washington, Seattle, WA, USA.; 8. Howard Hughes Medical Institute, Chevy Chase, MD, USA.

**Keywords:** single-cell genomics, CUT&Tag, sciCUT&Tag, ICELL8, histone modifications, epigenomic profiling, DNA sequencing

## Abstract

Cleavage Under Targets & Tagmentation (CUT&Tag) is an antibody-directed *in situ* chromatin profiling strategy that is rapidly replacing immune precipitation-based methods, such as ChIP-seq. The efficiency of the method enables chromatin profiling in single cells but is limited by the numbers of cells that can be profiled. Here, we describe a combinatorial barcoding strategy for CUT&Tag that harnesses a nanowell dispenser for simple, high-resolution, high-throughput, single-cell chromatin profiling. In this single-cell indexed CUT&Tag (sciCUT&Tag) protocol, lightly cross-linked nuclei are bound to magnetic beads and incubated with primary and secondary antibodies in bulk and then arrayed in a 96-well plate for a first round of cellular indexing by antibody-directed-Tn5 tagmentation. The sample is then repooled, mixed and arrayed across 5184 nanowells at a density of 12–24 nuclei per well for a second round of cellular indexing during PCR amplification of the sequencing-ready library. This protocol can be completed in two days by a research technician, and we illustrate the optimized protocol by profiling histone modifications associated with developmental gene repression (H3K27me3) as well as transcriptional activation (H3K4me1–2-3) in human peripheral blood mononuclear cells and use SNPs to facilitate collision removal. We have also used sciCUT&Tag for simultaneous profiling of multiple chromatin epitopes in single cells. The reduced cost, improved resolution and scalability of sciCUT&Tag make it an attractive platform to profile chromatin features in single cells.

## EDITORIAL SUMMARY

This protocol provides guidelines for performing sciCUT&Tag. This method builds on the existing CUT&Tag method and uses a combinatorial indexing step to allow for single-cell profiling of chromatin modifications.

## Introduction

### Development of the Protocol

Within the nucleus, genomic DNA is tightly packaged by histone octamers and DNA-binding proteins into chromatin, such that only a small minority of the genome is accessible to transcriptional machinery. Chromatin proteins and their modified states are the molecular determinants of protein-DNA interactions, and therefore scalable, genome-wide profiling of chromatin modifications is a powerful method to map the molecular mechanisms that govern not only transcription^1^, but also cell-fate specification^2^.

To quantify chromatin features genome-wide, we previously introduced Cleavage Under Targets and Tagmentation (CUT&Tag)^3^, a precipitation-free method that scales to single cells^4,5^. CUT&Tag uses a Tn5 cut-and-paste transposase fused to a protein A moiety (pA-Tn5), which tagments antibody-targeted genomic loci with PCR-ready adapters. As the Tn5 fusion protein remains bound to DNA following magnesium-catalyzed tagmentation, fragments are retained within intact cells, which makes CUT&Tag compatible with single-cell profiling. Single-cell resolution can then be achieved by partitioning the sample with either a droplet-based approach or nanowell dispenser and then introducing a cell-specific barcode by PCR that is read out during next-generation sequencing.

Since the advent of CUT&Tag, derivative methods have combined single-cell CUT&Tag with strategies for profiling cell features other than chromatin, such as mRNAs^6^ and cell-surface proteins^7^. This complementary information can then be used *a priori* to ascribe cell identity and generate a representative chromatin landscape of each cell type by aggregating the scCUT&Tag profiles. However, the genome-wide distributions of numerous chromatin modifications are tightly correlated with both tissue and cell type^8^. Thus, by obtaining sufficient data quality, unbiased high-resolution clustering and cell type identification is possible using the scCUT&Tag chromatin profiles alone. Here, we expand on the scCUT&Tag technology using a sample partitioning method called combinatorial indexing to dramatically increase the number of individual cells that can be profiled per experiment while maintaining precise control of the CUT&Tag reaction chemistry at each step. We provide a detailed description of the single-cell indexed CUT&Tag (sciCUT&Tag) protocol as performed on a nanowell dispenser. This sciCUT&Tag protocol is simple, highly reproducible, and is suitable for parallel profiling of multiple samples and epitope combinations in a single experiment. By multiplexing samples, sciCUT&Tag not only streamlines the data acquisition, but also facilitates data integration by limiting batch-to-batch variation and provides an opportunity to acquire biological replicates that power downstream statistical analysis. This sciCUT&Tag protocol was first used for the Multiple Target Identification by Tagmentation (MulTI-Tag)^9^ method that we recently introduced to profile multiple chromatin epitopes in individual cells. The high recovery of both cellular input and individual fragments in MulTI-Tag is enabled by an underlying single-cell partitioning method called combinatorial indexing, which facilitates the precise control of CUT&Tag reaction chemistry at each step.

Relative to droplet-based approaches, combinatorial indexing exponentially increases the number of single cells possible to profile per experiment for RNA-seq^10^, ATAC-seq^11^ and CUT&Tag^6,12,13^. Furthermore, combinatorial indexing presents the opportunity to optimize chemistry for fragment recovery without needing to also optimize chemistry for droplet formation. Through a single round of indexing, pooling, splitting and indexing, reads are labeled and then grouped by unique combinations of barcodes that statistically correspond to an individual cell of origin.

Two key variables can be scaled to increase the cellular yield from a combinatorial indexing experiment: number of split-pooling rounds and number of indices per round. However, scaling either or both increases the probability of technical error. Step-by-step protocols^14,15^ have therefore been introduced to streamline and standardize combinatorial indexing experiments. In the sciCUT&Tag protocol, we minimize the number of split-pooling rounds by increasing the number of indices per round from 96 to 5184 with the TaKaRa ICELL8 nanowell system^16^. We find that employing a robotic system for combinatorial indexing achieves a high degree of cell-per-well accuracy and tunability.

**Fig. 1:**
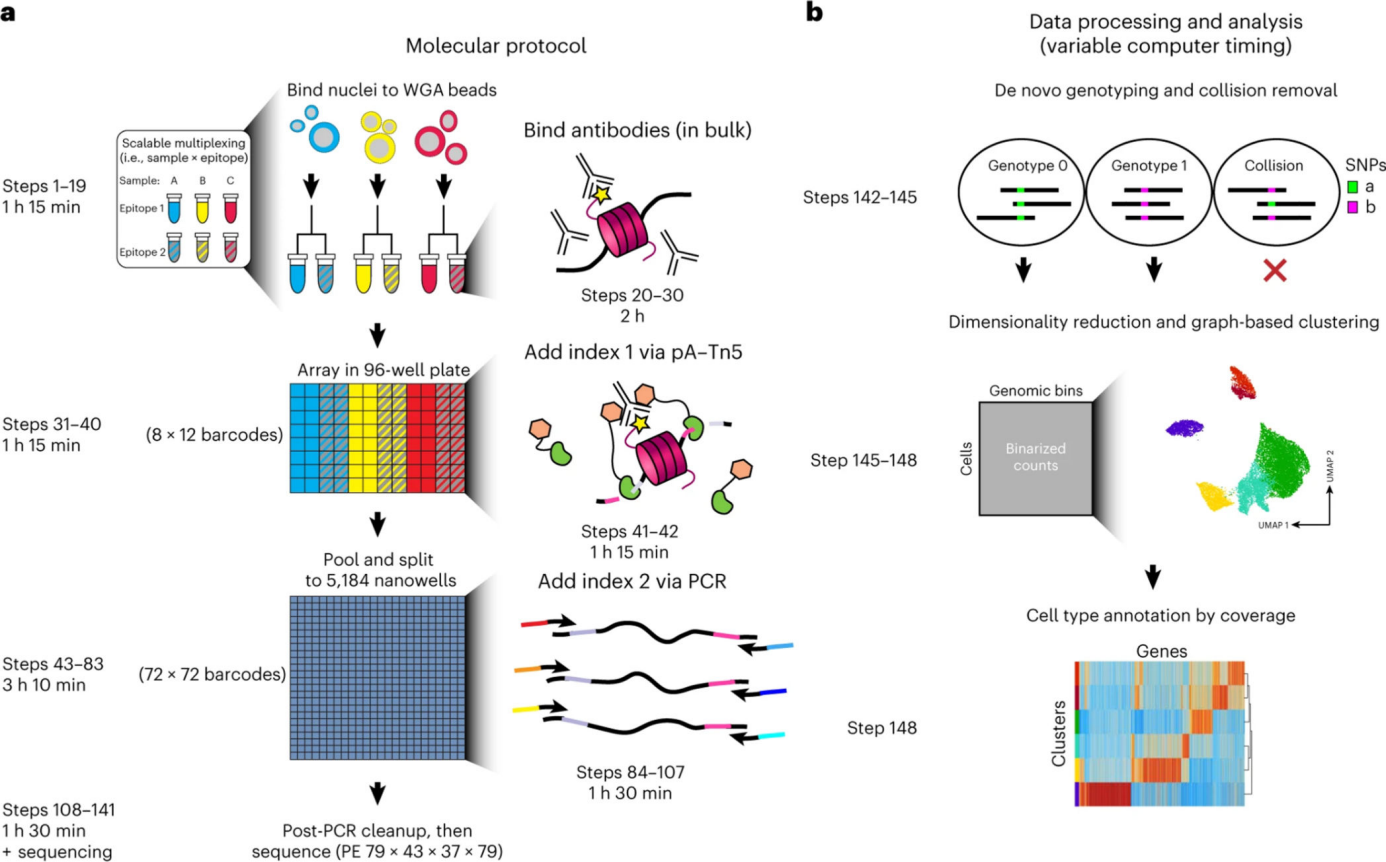
Overview of the sciCUT&Tag experimental workflow. a, Schematic of the sciCUT&Tag sample processing procedure. Cells are first incubated with primary and secondary antibodies in bulk, then arrayed in 96- well format for tagmentation with barcoded pA-Tn5. The 96-well plate is then pooled and split with a TaKaRa ICELL8 robot to the 5,184-well chip for PCR amplification that adds a second barcode before sequencing. Cells from different samples are indicated in blue, yellow and red; chromatin epitope conditions are hashed and unhashed; nucleosomes are colored fuschia with yellow star-shaped epitopes; pA-Tn5 is colored peach and green; barcoded indices are multicolored line segments next to black genomic template, b, Schematic of the informatics approach for sciCUT&Tag data processing. For mixed-donor samples, collisions are first detected by de novo genotyping to identify and remove barcodes with SNPs from multiple genotypes. Dimensionality reduction is then performed on a matrix of cells by genomic bins before graph-based clustering to identify subpopulations within a sample. These subpopulations than can then be assigned a celltype annotation based on gene coverage. Distinct SNPs at the same locus are green and pink within black cells. See text for details of the procedure.

sciCUT&Tag builds on the bulk CUT&Tag protocol^17^ in which nuclei are first isolated by a detergent wash and then immobilized on paramagnetic beads to facilitate supernatant removal while preserving yield (Fig. 1a). Incubation with primary and then secondary antibodies is then performed in bulk for each sample and epitope under investigation. The sciCUT&Tag workflow can accommodate simultaneous profiling of multiple samples, as individual sample/epitope combinations are then arrayed into a 96-well plate. Uniquely barcoded pA-Tn5 transposomes are then added to each well and tagmentation is performed in a 300 mM NaCl-containing buffer to add the first index *in situ*. In a streamlined combinatorial indexing paradigm, we perform only one subsequent round of indexing after pooling all nuclei from the 96-well plate and then splitting into a 5184-nanowell chip via a robotic device that facilitates reproducibility by reducing technical variation across experiments. Lastly, lysis is followed by PCR amplification to add the second index via barcoded primers in 5184-well format prior to sequencing.

To leverage informatics (Fig. 1b) for the removal of barcodes that correspond to ‘collisions’ where two nuclei stick together, we recommend designing sciCUT&Tag experiments to include samples with distinct genotypes that can be read out per cell to identify cross-genotype collisions. This incorporates a built-in quality control check, as certain tissues or cells may be ‘stickier’ than others. For sciCUT&Tag, we increase the sequencing read length relative to bulk CUT&Tag, as the increased genomic information facilitates deconvolution of multiple genotypes *de novo*. Barcoded read groups that contain multiple overlapping single-nucleotide polymorphisms (SNPs) are then removed prior to downstream analysis with dimensionality reduction, graph-based clustering, and cell type annotation.

### Applications of the method

In the development of this protocol, we first optimized sciCUT&Tag single-cell acquisition parameters by profiling a mixture of human and mouse cell lines. As an example of the protocol, we applied sciCUT&Tag to profile a mixture of human peripheral blood mononuclear cells (PBMCs) from two healthy donors with (1) an antibody cocktail that recognizes histone H3 lysine 4 mono-, di-, and tri- methylation (H3K4me1–2-3) and labels transcriptionally active promoters and enhancers, and with (2) a single monoclonal antibody that recognizes histone H3 lysine 27 trimethylation (H3K27me3), which labels Polycomb-associated, developmentally repressed chromatin. We show that single cell profiles of either chromatin modification are sufficient to perform high-resolution clustering and *de novo* identification of the cell types found in human PBMCs. In addition, we previously used the multifactorial variation of sciCUT&Tag, MultiTag, to resolve cells along a trilineage differentiation from human embryonic stem cells to definitive endoderm, mesoderm or neuroectoderm^9^.

In principle, sciCUT&Tag can be applied to any biological sample that is compatible with the isolation of lightly cross-linked nuclei. For example, this sciCUT&Tag platform is well suited for profiling patient samples to identify rare cell types or cell states associated with disparate clinical outcomes. The sciCUT&Tag method is comparable to single-cell RNA-seq and ATAC-seq methods that have facilitated comparative genomic analysis of human blood samples to identify gene regulatory changes in response to inflammatory stresses, such as those induced by COVID-19 infection, as well as inter- and intra-tumoral heterogeneity^18–20^. This sciCUT&Tag platform will enable investigators to expand these comparative genomic analyses to include other critical aspects of the epigenome, including regions maintained in developmentally repressed states.

### Comparison with other methods

Improvements have been made to sciCUT&Tag since it was first introduced^9^ that increase both reads/cell as well as cell recovery. Compared to other single-cell CUT&Tag approaches^4–7,12,13,21,22^, sciCUT&Tag offers at least a four-fold increase in throughput. Importantly, the sciCUT&Tag method drastically reduces the cost per sample via scalable multiplexing. With optimal loading conditions across an entire experiment (12–24 cells/nanowell), we estimate that sciCUT&Tag achieves a consistent yield of ~40,000 cells/chip, which corresponds to ~0.11 USD per cell in library preparation and sequencing costs. In comparison, standard droplet-based kits currently cost ~0.85 USD per cell, while obtaining fewer unique reads per cell than this sciCUT&Tag platform. Because each ICELL8 nanowell chip costs only 600 USD, it is cost-efficient to dispense the same pool of tagmented nuclei multiple times to generate additional sequencing libraries and capture more unique single-cell profiles per experiment. The sciCUT&Tag method also represents a near doubling in reads/cell versus our initial scCUT&Tag^5^ method (2116 versus 1110 median reads/cell, respectively, for H3K27me3 in human PBMCs) and is comparable to methods that use linear amplification-based library preparation to bolster reads/cell^12,21^.

### Overview of the procedure

The sciCUT&Tag method for single-cell chromatin profiling can be performed on the benchtop and completed in 1.5 days using the TaKaRa ICELL8 robotic nanowell system. In brief, lightly cross-linked nuclei are prepared and immobilized on wheat germ agglutinin (WGA)-coated paramagnetic beads (Steps 1–19; Fig. 1a)^23^. Bead-bound nuclei are incubated with a primary antibody followed by incubation with a secondary antibody to increase the number of IgG molecules at each epitope bound by the primary antibody (Steps 20–30; Fig. 1a). Bead-bound nuclei are washed and arrayed in a 96 well plate for incubation with protein A-Tn5 loaded with differentially barcoded mosaic-end adapters and washed under stringent conditions (Steps 31–40; Fig. 1a). Tn5 is activated by addition of Mg^2+^, and tagmentation is carried out at 37 °C (Steps 41–42; Fig. 1a). Tagmented bead-bound nuclei are repooled, washed and passed through a 20 μm Mini-Strainer to remove cellular aggregates, and are then dispensed on the ICELL8 into a 5184 350v chip, imaged and prepared for PCR in 0.19% SDS Release buffer (Steps 43–83; Fig. 1a). Triton-X neutralizing solution and PCR reagents are added through a series of dispenses on the ICELL8 and sciCUT&Tag libraries are enriched by PCR amplification (Steps 84–107; Fig. 1a) and two Solid Phase Reversible Immobilization (SPRI) magnetic bead cleanup steps (Steps 108–125; Fig. 1a). The sciCUT&Tag library is quantified on an Agilent TapeStation capillary gel analyzer and diluted for sequencing on an appropriate flow cell as determined by the initial library concentration (Steps 126–141; Fig. 1a). After sequencing, data processing steps142–148 demultiplex samples according to the four-barcode combinatorial indexing scheme using the sciEXtract custom code, align fastq files to the genome of interest and remove doublets using SNP-based detection or using a nearest neighbor approach with synthetic doublets. Dimensionality reduction and clustering is performed and annotated based on distribution of chromatin features surrounding marker genes.

### Experimental Design

For sciCUT&Tag, we recommend designing experiments to include multiple samples with distinct genotypes, as this helps detect ‘collisions’ where a barcode combination corresponds to more than one cell. As few as two samples can be mixed prior to immobilization on paramagnetic beads for this approach. **To keep nuclei intact and to reduce the formation of multi-nuclear aggregates,** light cross-linking is critical.

While multiple epitopes can be profiled in parallel with sciCUT&Tag^9^, the most abundant epitopes should be prioritized to increase reads/cell yield, which is essential for dimensionality reduction during data analysis. The number of nuclei per antibody can vary greatly depending on the experimental design. For example, for an experiment in which two different histone modifications will be profiled in an equal number of nuclei, the sample should be split evenly in two tubes and processed in parallel and allocated a corresponding proportion of the 96-well plate for sci-pA-Tn5 binding and tagmentation. Pooling antibodies may also be used to garner sufficient analyzable signal for less frequent epitopes, and we expect that antibodies validated by the manufacturer for CUT&RUN or immunofluorescence are most likely to work for this application. Throughout the protocol, we recommend incubating the samples with a relatively high concentration of the primary antibody (1:10 dilution by volume) and secondary antibody (1:20 dilution by volume) as well as the sci-pA-Tn5 complexes (1:10 dilution by volume) in order to saturate the binding of the chromatin protein of interest and increase the number of unique reads obtained per cell. We have found the secondary antibody step is required for CUT&Tag to increase the number of Protein A binding sites for each chromatin target. Without the secondary antibody, the efficiency of tagmentation is very low. The immunoglobin-binding moiety of pA-Tn5 binds tightly only to certain IgG isotypes of some host species, for example rabbit and guinea pig IgG, but poorly to mouse IgG_1_ and goat IgG. Thus, the secondary amplifying antibody must be chosen to provide high affinity for protein-A. For example, for mouse primary antibodies we recommend a rabbit anti-mouse antibody be used.

In order to increase the number of single cell profiles captured in each experiment, the second round of indexing is performed using the ICELL8 nanowell dispenser. Because the bead-bound nuclei slowly sink, which can cause the ICELL8 chip to be loaded unevenly, we use the “Pause and Dispense protocol”, available upon request from TaKaRa technical support. This custom program will load the negative control and then pause three times during the cell dispense process to allow for sample mixing immediately prior to aspiration of the sample solution by the ICELL8.

During sciCUT&Tag library sequencing, the length of the barcode reads is fixed and read out using 43 cycles for the second read and 37 cycles for the third read. The length of the genomic reads can be variable depending on the application. When mixing samples from multiple donors for SNP-based doublet detection, it is important to obtain sufficient genomic information to detect donor specific SNPs. For this we have used either the Next-Seq P2–200 flow cell, which yields 79 bp by 79 bp paired ends (79 × 43 × 37 × 79) or the Next-Seq P1–300 flow cell, which yields 129 bp by 129 bp paired ends (129 × 43 × 37 × 129).

## Example validation of the method

To optimize our sciCUT&TAG protocol, we identified split-pooling and sample loading conditions that minimize the number of cellular collisions arising from two or more cells receiving the same combination of barcodes, and also empirically determined the H3K4me1–2-3 and H3K27me3 modification specific parameters for *in silico* dimensionality reduction that robustly separate the major cell types found in human peripheral blood. These steps were necessary for development of the protocol, but most users can follow the guidelines here and do not need to repeat optimization experiments.

### Loading conditions for the ICELL8 5184-well chip

We first estimated the rate of ‘collisions’ by overloading the ICELL8 5184-well chip in a ‘barnyard’ experiment that mixed human and mouse cell-lines at a 1:1 ratio and dispensed them across a serial dilution of 40 to 10 cells/nanowell in duplicate (Supplementary Fig. 1a and b). Collisions were called as barcodes with fewer than 90% of reads uniquely aligned to either hg38 (human) or mm10 (mouse). We found that collisions dominated the low-content barcodes with fewer than 500 reads (Supplementary Fig. 1c; grey box). This allowed us to set a low threshold of at least 500 reads (Supplementary Fig. 1c; **black dashed line**) for a barcode to be called as a single cell. We excluded these low-content barcodes when calculating the rate of collision across the serial dilution of dispense targets (Supplementary Fig. 1d). For the dispense target of 40 nuclei/nanowell, we observed the highest collision rate of 21.2%; and this condition was also estimated to have the highest yield at just under 40,000 cells/chip. In line with the serial dilution, cells/chip yield decreased ratiometrically with the dispense target and the rate of collisions also decreased in accordance with the number of nuclei dispensed per nanowell. Specifically, at 20 nuclei/nanowell the rate of collisions was 17.7%, at 10 nuclei/nanowell the rate of collisions was 16.0%, and at 5 nuclei/nanowell the rate of collisions fell below 12%. We conclude that the number of nuclei loaded per nanowell must be carefully controlled to limit collisions and obtain an optimum number of single-cell profiles per experiment.

As expected from overloading the barnyard experiment, we observed that barcodes/well across both the 96-well plate and 5184-well chip highly correlated with low-content barcodes and collisions (Supplementary Fig. 1c and d). Given that nuclei are barcoded by well in combinatorial indexing, a well that is overloaded in the 96 well plate during the first round of indexing will give rise to a higher frequency of collisions during the second round of indexing. In addition, overloading may cause the tagmentation and/or PCR chemistry to become rate-limiting, which can potentially reduce reads/barcode and weaken analytical power during dimensionality reduction. Indeed, we observed that that one well in the 96-well plate gave rise to 3126 of the barcodes recovered by sequencing, the majority of which had either low content or were a collision (Supplementary Fig. 1a). Furthermore, we observed that barcodes/well strongly correlated with low-content cells/well across both the 96-well plate (Pearson’s *r* = 0.99) and 5184-well chip (Pearson’s *r* = 0.93) (Supplementary Fig. 1e and f). Concordantly, collisions/well also exhibited a high correlation with barcodes/well across both the 96-well plate (Pearson’s *r* = 0.90) and 5184-well chip (Pearson’s *r* = 0.63).

While this pattern was maintained when correlating barcodes/well and collisions with high-content cells/well (e.g., barcodes with >500 reads) for the 96-well plate (Supplementary Fig. 1g; Pearson’s *r* = 0.91 and 0.88, respectively), it was not well-maintained for the 5184-well chip (Supplementary Fig. 1h; Pearson’s *r* = 0.43 and 0.49). This suggests two important conclusions: (1) that events which produce low-content barcodes and collisions originate in the 96-well plate, and (2) that the nanowells which yield the most cells above threshold are distinct from those which yield low-content barcodes and collisions. While overloaded chemistry in the 5148-cell chip can account for the second observation, possible explanations for the first observation are that clumping and/or lysis in the 96-well plate can increase total barcode count at the expense of low-content barcodes and collisions. To limit clumping and lysis, we emphasize careful pipetting with wide-bore tips and filtering the pooled tagmentation product with a 20 μm Mini-Strainer prior to dispense on the ICELL8.

**Fig. 2:**
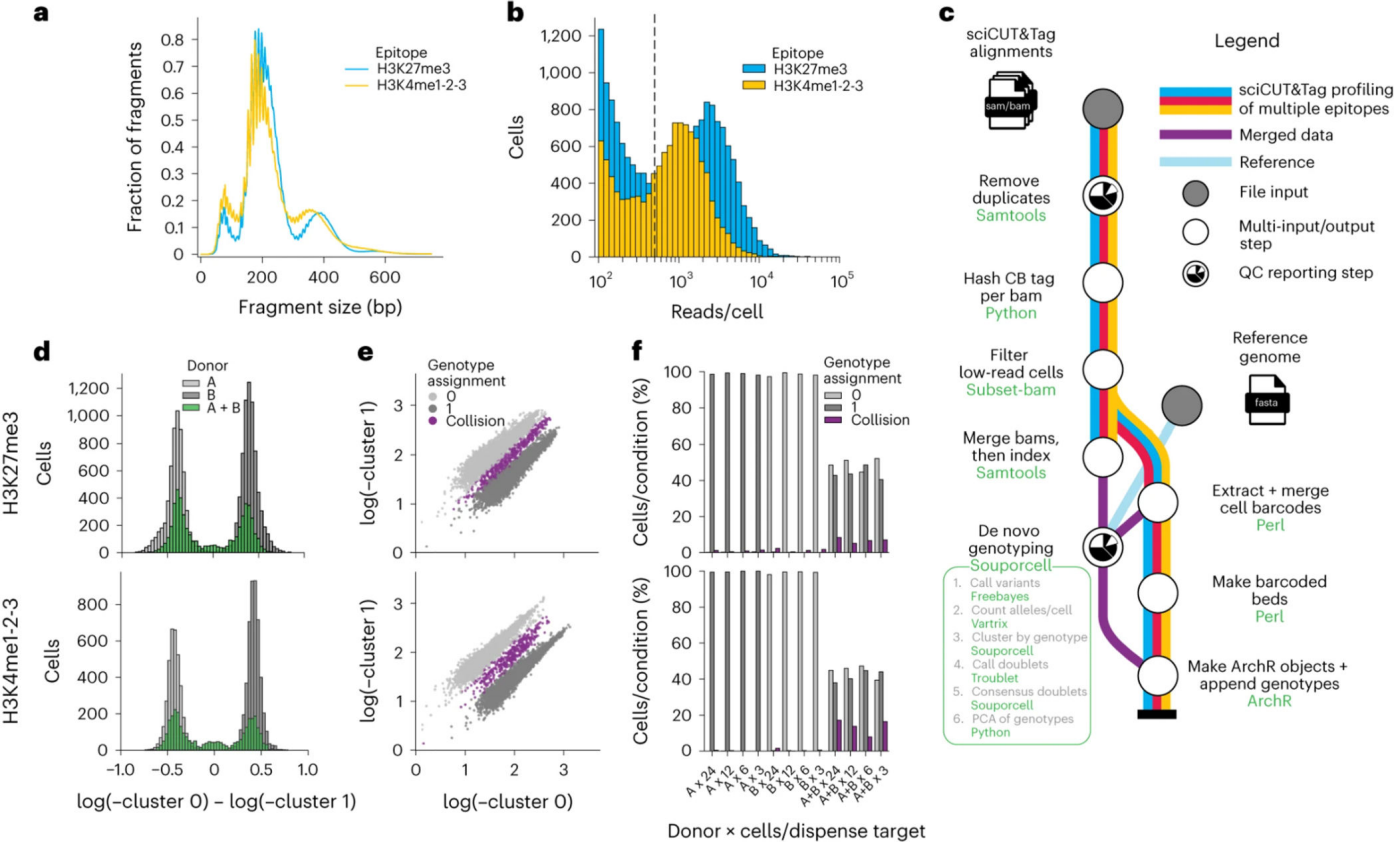
Validation of a de novo genotyping strategy for SNP-based collision removal in sciCUT&Tag of mixed-donor PBMCs. a, Fragment size distributions for H3K27me3 (blue) and H3K4mel-2–3 (yellow) sciCUT&Tag profiles, b, Reads/cell distributions for H3K27me3 (blue) and H3K4mel-2–3 (yellow) sciCUT&Tag profiles. A black-dashed line marks 500 reads/cell and barcodes with less than 500 reads are excluded from further analyses, c, Nextfloŵ pipeline schematic built on Souporcell^ for de novo genotyping and SNP-based collision removal. sciCUT&Tag profiling of multiple epitopes are the blue, red and yellow paths that are merged into one purple path before de novo genotyping with Souporcell. Input files are genomic alignments and the reference genome used for alignment. The cell barcode is expected in the read name and is parsed to the cell barcode (CB) tag of the genomic alignment. Outputs are barcoded bed files, PCA of Souporcell cluster assignment probabilities, an ArchR^ object annotated with genotype assignments and tabulated cell metadata. The software used in the pipeline are shown in green and the pipeline terminus is a black line, d, Cell counts versus the log differential of the genotype assignment cluster probabilities colored by donor for H3K27me3 (top) and H3K4mel-2–3 (bottom), e, The two genotype assignment cluster probabilities plotted against each other and colored by genotype assignment for H3K27me3 (top) and H3K4mel-2– 3 (bottom), f, The percentage of cells per condition (donor x dispense target) for each genotype assignment for H3K27me3 (top) and H3K4mel-2–3 (bottom).

Given the possibility of high collision frequency in combinatorial indexing experiments, we implemented a collision removal strategy based on *de novo* genotyping that can be widely applied to any tissue or cell line for which biological replicates with distinct genotypes can be obtained. As little as two samples can be mixed prior to immobilization on paramagnetic beads for this strategy and multiple epitopes can be simultaneously profiled in parallel in a single sciCUT&Tag experiment. We demonstrated this scalable multiplexing approach by mixing PBMCs from two donors and profiling both H3K27me3 and H3K4me1–2-3 across the mixed sample as well as the single donor samples representing ground truth. Both H3K27me3 and H3Kme1–2-3 profiles exhibited classic 146 base-pair nucleosomal laddering (Fig. 2a), and we recovered a greater number of reads/cell above the low-content threshold of at least 500 reads/cell (Fig. 2b; **black dashed line**) for H3K27me3 (μ = 2649 reads/cell) than H3K4me1–2-3 (μ = 1420 reads/cell).

We next built on the Souporcell^24^ tool for identifying cross-genotype collisions based on single nucleotide polymorphisms (SNPs) in mixed donor samples, integrating it into an easy-to-use and reproducible Nextflow pipeline^25,26^. The pipeline combines sciCUT&Tag genome alignments for multiple epitopes into a single Souporcell run to maximize sequence space for *de novo* genotyping, and then outputs barcoded bed files, principal component analysis (PCA) of Souporcell cluster assignment probabilities, and tabulated cell metadata (Fig. 2c). This collision removal strategy may be insufficient to distinguish two unique genotypes from sciCUT&Tag profiles generated by paired-end 25 × 25 or 50 × 50 base sequencing, so we increased the read length to paired-end 79 × 79.

We benchmarked the pipeline in the mixed-donor PBMC experiment by first counting the cells assigned to each genotype and mapping them to their donor of origin. Indeed, for both the H3K27me3 and H3K4me1–2-3 profiles, Donor A associated with genotype 1 and Donor B associated with genotype 0 (Fig. 2d). As expected, the mixed donor population was evenly distributed across both genotype assignments with only a small intermediate population. Because only two donors were mixed, we were able to plot the cluster assignment probabilities against each other instead of by PCA, which revealed that genotypes 0 and 1 assignments neatly separated, and collisions aligned with the small intermediate population (Fig. 2e). The collision population also scored significantly higher than single-genotype cells for DoubletEnrichment in the artificial nearest neighbor (ANN) approach implemented in ArchR^27^, which corroborated their mixed-phenotype character (Supplementary Fig. 2).

Including the single-donor ground truth samples in addition to the mixed donor sample provided us with a comprehensive basis to assess the accuracy of Souporcell^24^ for identifying cross-genotype collisions in sciCUT&Tag (Fig. 2f). Indeed, we observed nearly zero collisions in the single-donor ground truth samples across dispense targets. Compared to H3K27me3 (μ = 7.0% collision rate; max = 8.5%), H3K4me1–2-3 exhibited a higher percentage of collisions across the serial dilution (μ = 13.0%; max = 17.2%). Together, efficient combinatorial indexing on ICELL8 system augmented by *de novo* genotyping and SNP-based collision removal comprise a robust approach to single-cell chromatin profiling in sciCUT&Tag. Importantly, while we were able to obtain a ground truth of cellular collisions using Souporcell, the accuracy of SNP-based doublet callers is highly dependent on the data quality, including the number of reads obtained per cell, the genetic diversity of the donor samples to be mixed, the length of the genomic reads and the fraction of reads that overlap one another to allow SNP comparisons. So, while Souporcell is a viable approach for calling doublets in H3K4me1–2-3 and H3K27me3 sciCUT&Tag data, whether this extends to sciCUT&Tag profiles of other epitopes must be determined empirically.

### Tailoring dimensionality reduction to the biology of different chromatin epitopes.

The advantage of single-cell approaches like sciCUT&Tag over bulk CUT&Tag are that they enable identification of cell populations within heterogenous samples. By parsing sparse chromatin profiles with unsupervised dimensionality reduction, latent representations are revealed that drive clustering of like cells for downstream analysis. Across single-cell analyses, the input to dimensionality reduction algorithms is a cell-by-feature matrix, and the output is a latent representation with a condensed number of dimensions/cell that can be further reduced to two for visualization with algorithms such as uniform manifold approximation and projection (UMAP)^28^. To identify the optimal dimensionality reduction parameters for different types of chromatin, such as variably phased heterochromatic epitopes that form broad domains and tightly positioned nucleosomes at promoters, we tested a range of cell-by-genomic-bin feature representations for H3K27me3 and H3K4me1–2-3 profiling in PBMCs, which we expected to separate into distinct immune cell types based on previous analysis^5^.

**Fig. 3:**
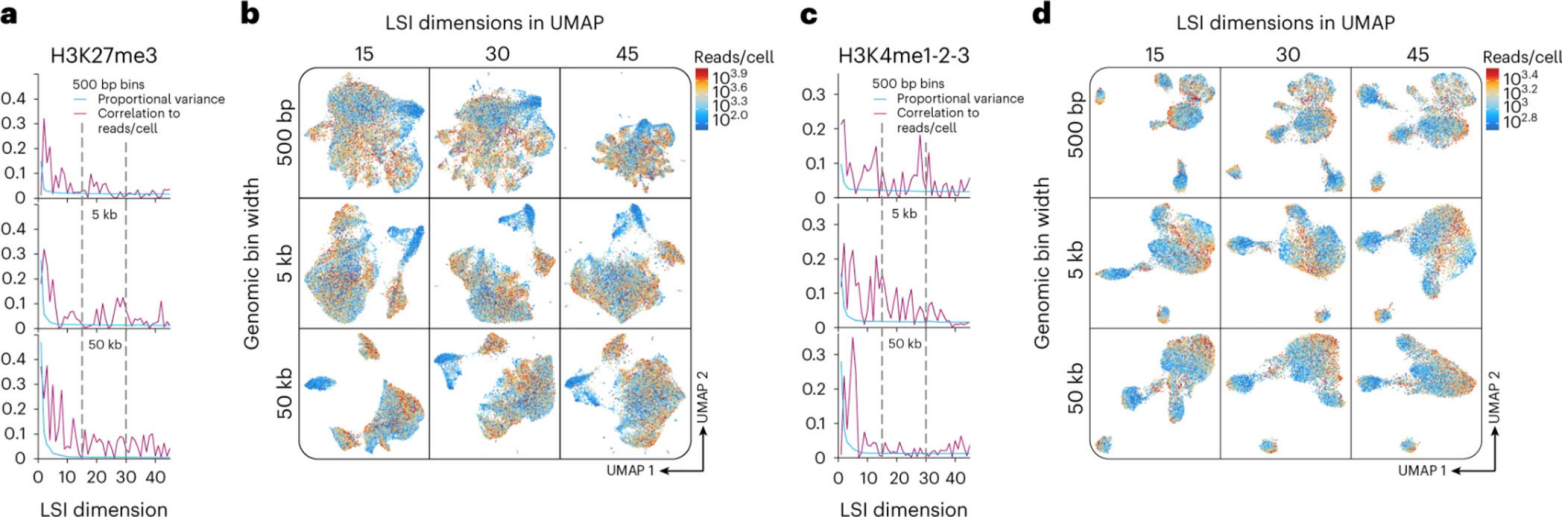
Systematic investigation of dimensionality reduction parameters for sciCUT&Tag. a, H3K27me3 profiling in PBMCs was binned at 500 bp, 5 kb and 50 kb genomic intervals, then input to iterative LSI— for dimensionality reduction. Proportional variance (blue) and correlation to reads/cell (red; Pearson’s *r)* was calculated across the first 45 LSI dimensions. Gray lines mark dimensions 15 and 30. b, For H3K27me3, UMAPs were computed from the first 15,30 and 45 LSI dimensions and colored by reads/cell, c, H3K4mel-2–3 profiling in PBMCs was binned at 500 bp, 5 kb and 50 kb genomic intervals, then input to iterative LSI for dimensionality reduction. Proportional variance (blue) and correlation to reads/cell (red; Pearson’s *r*) was calculated across the first 45 LSI dimensions. Gray lines mark dimensions 15 and 30. d, For H3K4mel-2–3, each bin width, UMAPs were computed from the first 15,30 and 45 LSI dimensions and colored by reads/cell.

To systematically optimize dimensionality reduction of sciCUT&Tag data, we used the iterative latent semantic indexing (LSI) approach implemented in ArchR^27^ to reduce matrices with 500 bp, 5 kb, and 50 kb genomic bins. Information content and noise for the first 45 dimensions of LSI was estimated for each binning paradigm as proportional variance and correlation to reads/cell, respectively. As a realistic next step for data processing, we then computed UMAPs for the first 15, 30, and 45 LSI dimensions and shaded them by reads/cell to check for embedding bias. For the repressive epitope H3K27me3, we found that increasing genomic bins to 50 kb pushed nearly all variance into early LSI dimensions, while correlation to reads/cell remained distributed into late dimensions (Fig. 3a). Concordant with the low variance in late dimensions, inputting more than 15 dimensions to UMAP did not improve separation across genomic bin widths (Fig. 3b). For H3K27me3, these findings advocate for representation of broad domains by larger genomic intervals.

While increasing bin width may improve dimensionality reduction for broad H3K27me3 domains, it can reduce resolution by merging distinctly regulated loci in larger features. For H3K4 methylation that adorns well-phased nucleosomes in active domains like transcriptional start sites (TSSs), smaller bins can maximize feature resolution. Indeed, for H3K4me1–2-3, unlike for H3K27me3, we found that increasing genomic bins to 50 kb did not increase early dimension variance, but rather increased correlation to reads/cell in early LSI dimensions (Fig. 3c), and polarized UMAP embeddings by reads/cell (Fig. 3d). In contrast, we observed the best UMAP separation for H3K4me1–2-3 with 500 bp bins and 15 LSI dimensions. Not only does this confirm that fewer dimensions are sufficient for UMAP embedding of sparse chromatin data, but also that smaller genomic intervals are better suited to represent narrow chromatin domains, such as those marked by H3K4 methylation.

## Limitations

The high throughput that we obtain requires the 72×72 nanowell chip used in the ICELL8 robotic system, which is not as widely available as droplet systems. However, the strategy described here can be carried out in microtiter plates. For example, using a 384-well plate for the split in place of the 5184-nanowell chip will provide up to ~4000 single cells.

As in bulk CUT&Tag, tagmentation during sciCUT&Tag is performed under 300mM NaCl to limit off-target localization by pA-Tn5 to accessible DNA. While high-salt conditions ensure accurate tagmentation at the targeted epitope, they also competitively reduce occupancy of transcription factors (TFs), making sciCUT&Tag best for higher-affinity targets, such as histone modifications. Of note, histone modifications are also the preferred epitopes for single-cell profiling because of their high abundance in most cell types, and it is rare for single-cell methods to be applied to TFs because of their low abundance, which limits the power of dimensionality reduction. Bulk CUT&RUN remains the preferable method for both base-pair resolution and high signal-to-noise profiling of TFs.

## MATERIALS

### Biological materials

- Cell lines. We have used human K562 cells (ATCC; CCL-243; https://scicrunch.org/resolver/RRID:CVCL_0004), H1 cells (WiCell; WA01-lot#WB35186; https://scicrunch.org/resolver/RRID:CVCL_9771), mouse 3T3 cells (https://scicrunch.org/resolver/RRID:CVCL_0594) and other mammalian cell lines, as well as healthy human peripheral blood mononuclear cells (PBMCs). A description of the PBMC acquisition and processing is provided in the Supplementary methods.
  **CAUTION:** The cell lines used in your research should be regularly checked to ensure they are authentic and are not infected with mycoplasma. When working with primary human samples ensure the study complies with all relevant governmental and institutional review boards, and that the informed consent is obtained from all study participants.

### Reagents

- 3XFlag-pA-Tn5-Fl (pA-Tn5) plasmid (Addgene, cat. no. 124601)
- 5 mL of purified Protein A–Tn5 (pA-Tn5) fusion (https://www.protocols.io/view/3xflag-patn5-protein-purification-and-meds-loading-j8nlke4e5l5r/v1). This is sufficient to prepare a loaded 96-well sci-pA-Tn5 for up to 30 experiments.
- Tn5 Mosaic-End Adapters: 1 universal MED-REV oligo as well as 8 s5 adapter oligos and 12 s7 adapter oligos with unique barcodes are listed in Supplementary Table 1. We ordered s5 and s7 oligos from IDT prepared by PAGE purification on a 1 μmole scale.
- PCR primers: 72 i5 PCR primers and 72 i7 PCR primers with unique barcodes adapted from Buenrostro et al^29^ are listed in Supplementary Table 1. We order i5 and i7 PCR primers from IDT, in salt-free format on a 25 nmole scale; higher purification formats are optional. Our PCR conditions are optimized for these custom primers.
  **CAUTION:** Do not use Nextera primers, which will not work efficiently.
- Custom sequencing primers: 4 Custom oligos for sequencing the sciCUT&Tag library on an Illumina NextSeq 2000 instrument are listed in Supplementary Table 1. We order these primers from IDT using standard desalting conditions on a 25 nmole scale.
- Nuclease-free H_2_O (Promega, cat. no. P1197)
- HEPES Sodium Salt (Sigma-Aldrich, cat. no. H3375)
- 1 M Potassium Chloride (KCl; Sigma-Aldrich, cat. no. P3911)
- 10% Triton X-100
- 2 M Spermidine (Sigma-Aldrich, cat. no. S2501)
- Glycerol (Sigma-Aldrich, cat. no. G5516)
- Roche Complete Protease Inhibitor EDTA-Free tablets (Sigma-Aldrich, cat. no. 5056489001)
- 10X Phosphate Buffer (PBS; Fisher Scientific cat. no. BP3991)
- Tween 20 (Sigma-Aldrich, cat. no. 274348–500mL)
- Pierce 16% Formaldehyde (w/v), Methanol-free (Fisher Scientific cat no. 28906)
  **CAUTION:** Formaldehyde is a cross-linking reagent that is hazardous if swallowed, inhaled, or absorbed through the skin. Proper PPE should be worn, and the workspace should be adequately ventilated. Preferably keep the formaldehyde solution in a fume hood to avoid inhalation.
- 0.5 M Ethylenediaminetetraacetic acid (EDTA; Research Organics, cat. no. 3002E)
- 5 M Sodium chloride (NaCl; Sigma-Aldrich, cat. no. S5150–1L)
- 5 mg/mL 4’, 6-Diamidino-2-phenylindole (1000X DAPI aliquots; Sigma-Aldrich, cat. no. D9542–50 mg)
- Dimethyl sulfoxide (DMSO; Sigma-Aldrich cat. no. D4540)
- Dynabeads^™^ MyOne^™^ Streptavidin C1 (Invitrogen, cat. no. 65001). Alternatively, Magnefy^™^ Concanavalin A ready-to-use beads (Bangs Laboratories, Inc. cat no. MFY531) can be used in the same way.
- Wheat Germ Agglutinin (WGA), Biotinylated (Vector Laboratories, Inc., cat. no. B-1025–5)
- 1 M Manganese Chloride (MnCl_2_; Sigma-Aldrich, cat. no. 203734)
- 1 M Calcium Chloride (CaCl_2_; Fisher, cat. no. BP510)
- 1 M Potassium Chloride (KCl; Sigma-Aldrich, cat. no. P3911)
- 100 mM Magnesium Chloride (MgCl_2_; Sigma-Aldrich, cat. no. M8266–100G) 3002E)
- TAPS (Millipore Sigma, cat. no. T5130–25G)
- 10% Sodium dodecyl sulfate (SDS; Sigma-Aldrich, cat. no. L4509)
- KAPA HiFi PCR Kit with dNTPs (Roche, cat no. 07958838001)
- Solid Phase Reversible Immobilization (SPRI) magnetic beads (e.g. Agencourt AMPure XP, Beckman Coulter, cat. no. A63880)
- 1 M Tris-HCl pH 8.0 (Fisher cat no. BP1521)
- Ethanol (Decon Labs, cat. no. 2716)
- Qubit dsDNA HS kit (Life Technologies, cat. no. Q32851)
- Illumina NextSeq Sequencing Reagents (e.g. P2 Reagents 200 cycles)

#### Antibodies

- Primary antibody to chromatin protein
  **CRITICAL:** High abundance epitopes such as histone modifications are optimal to obtain sufficient reads per cell and produce meaningful separation of cell types and cell states during downstream analysis. For example validation of this protocol, we used Anti-H3K27me3 rabbit monoclonal antibody (Cell Signaling Technology, cat. no. 9733); https://scicrunch.org/resolver/RRID:AB_2616029), anti-H3K4me1 rabbit oligoclonal antibody (Thermo, cat. no. 710795; https://scicrunch.org/resolver/RRID:AB_2532764), and anti-H3K4me2 rabbit polyclonal antibody (Millipore, cat. no. 07–030; https://scicrunch.org/resolver/RRID:AB_310342) primary antibodies.
- Secondary antibody to primary antibody
  **CRITICAL:** The secondary antibody must recognize the host type of the primary antibody and is necessary to increase the number of IgG molecules per targeted epitope. This increases the nucleation of pA-Tn5 at antibody targeted regions of the genome to ensure robust tagmentation. For rabbit primary antibodies we recommend a guinea pig anti-rabbit (Antibodies-Online ABIN101961; https://scicrunch.org/resolver/RRID:AB_10775589). For mouse primary antibodies we recommend a rabbit anti-mouse secondary antibody (e.g. Abcam ab46540; https://scicrunch.org/resolver/RRID:AB_2614925)

#### Cell Culture Reagents

- Matrigel (Corning cat. no. CLS354234)
- mTeSR1 Basal Media (STEMCELL Technologies cat. no. 85851)
- mTeSR1 Supplement (STEMCELL Technologies cat. no. 85852)
- RPMI 1640 Medium (ThermoFisher cat. no. 11875093)
- Glutamine and HEPES supplement (ThermoFisher cat. no. 72400047)
- FBS (ThermoFisher cat. no. 16141079)
- DMEM + Glutamax (ThermoFisher cat. no. 10566016)
- Gibco Antibiotic-Antimyocotic (Fisher Scientific cat. no. 15–240-062)

## EQUIPMENT

- ICELL8 single-cell systems (TaKaRa)
- ICELL8 350v chips (TaKaRa, cat. no. 640019)
- SMARTer ICELL8 Blank Chip Reagent Kit (TaKaRa Bio USA, cat. no. 640196)
- SMARTer ICELL8 Loading Kit (TaKaRa Bio USA, cat. no. 640109)
- SMARTer ICELL8 Collection Kit (TaKaRa Bio USA, cat. no. 640048)
- Countess 3 Automated Cell Counter (ThermoFisher Scientific)
- T100 thermal cycler (BIORAD cat. no. 1861096)
- 96S Super Magnet Plate (Alpaqua cat no. A001322)
- Centrifuge Eppendorf 5810, swinging bucket
- MicroAmp Support Bases (ThermoFisher, cat. no. N801–0531)
- Centrifuge Eppendorf 5424, fixed angle rotor
- Centrifuge Eppendorf 5415R, refrigerated fixed angle rotor
- Macsimag magnetic separator (Miltenyi, cat. no. 130–092-168).
- Vortex mixer (e.g., VWR Vortex Genie)
- Micro-centrifuge (e.g., VWR Model V)
- Nutator (e.g., Corning cat. no. 6720)
- pH Meter (e.g., Thermo Scientific cat. no. VSTAR83)
- Cell culture incubator (Sanyo cat no. MCO-19AIC)
- Conical centrifuge tubes (15 mL or 50 mL)
- Cryogenic screw-cap vials (Corning cat. no. 430658)
- Reagent Reservoirs (e.g. Thermo Scientific cat. no. 8095)
- AlumaSeal 96 film (e.g., Sigma Aldrich cat. no. Z721549–100EA)
- Mr. Frosty containers (Thermo cat. no. 5100–0001)
- 96 well LoBind PCR plates Semi-skirted (Eppendorf, cat. no. 0030129504)
- 0.65 mL Prelubricated microcentrifuge tube, DNase/RNAse free (Costar, cat. no. 3206)
- 1.5-mL microcentrifuge tubes (Genesee, cat. no. 22–282)
- 1.7-mL Microtube, Low Retention, DNase/RNAse free (Genesee Scientific, cat. no. 24–282LR)
- Mini-Strainer 20 μm (PluriSelect USA, cat. no. 43–10020-40)
- 2-mL microcentrifuge tubes (Axygen, cat. no. MCT-200-C)
- Capillary electrophoresis instrument (e.g. Agilent Tapestation 4200)
- 2–20 μL 8-Channel Multi Pepette (e.g. Rainin cat. no. 17013803)
- 20–200 μL 8-Channel Multi Pepette (e.g. Rainin cat. no. 17013805)
- 200 μL Wide Bore Tip Boxes (e.g., Rainin cat. no. 30389241)
- 5–50 μL 16-Channel Multi Pipette (e.g. CappAero cat. no. C50–16)
- Illumina NextSeq2000

### Software

- Souporcell (https://github.com/wheaton5/souporcell)
- Souper-star (https://github.com/FredHutch/souper-star)
- ArchR (https://github.com/GreenleafLab/ArchR)
- sciEXtract (https://github.com/mfitzgib/sciCTextract)

## REAGENT SETUP

### Cell culture

Cells of choice should be cultured or isolated before beginning the experiment. We have used human K562 cells, H1 human embryonic stem cells, ML-2 and RS4;11 cells, mouse 3T3 cells and PBMCs; culture and isolation conditions for these cells can be found in the Supplemental methods.

### Sci-pA-Tn5 Plate:

1. Anneal each of the 8 Barcoded Mosaic end - s5 adapters (ME-s5) and the 12 Barcoded Mosaic end - s7 adapters (ME- s7) oligonucleotides with Mosaic end – reverse oligonucleotides (Supplementary Table 1)^25^. To anneal, first dilute oligonucleotides to 200 μM in annealing buffer (10mM Tris pH8, 50mM NaCl, 1 mM EDTA). Mix 200 μM ME-s5 oligo with 200 μM ME-reverse oligo resulting in 100 μM ME-s5 adapter. Mix 200 μM ME-s7 oligo with 200 μM ME-reverse resulting in 100 μM ME-s7 adapter.
2. Place the tubes in a Thermocycler and run an annealing program with a 105°C heated lid that starts with a 95°C 2-minute incubation and decreases the temperature in 5°C increments incubating 5 minutes each, ending with a 5 minute incubation at 25°C and the hold at 8°C. Keep on ice for immediate use or store at −20°C. Annealed adapter stocks can be kept at −20 °C for at least a year.
3. Place 96 well LoBind PCR plate on ice and mix 3.2 μl of the appropriate preannealed ME-s5 adapter (100 μM) with 3.2 μl of the appropriate preannealed ME-s7 adapter (100 μM). Immediately add 40 μl of 5.5 μM pA - Tn5 fusion protein in 50% Glycerol, pipette up and down ~5 times to mix. An example of the Sci-pA-Tn5 Plate Layout is provided in Supplementary Table 2.
4. Incubate the mixture for 1 hour at room temperature (23–27 °C) and then store at −20 °C until use. The loaded sci-pA-Tn5 plate is stable at −20 °C for at least a year.
  **CRITICAL:** The volumes of ME-s5 adapter, ME-s7 adapter and the pA-Tn5 fusion can be scaled in proportion to one another. In addition, before loading an entire 96-well plate, it is advisable to perform a serial dilution experiment adjusting the ratio of the ME-s5 and ME-s7 adapter mixture to the pA-Tn5 to ensure the loading conditions are optimized for a given pA-Tn5 prep. The optimum loading condition can then be determined empirically by running parallel samples using the various pA-Tn5 preps. The relative efficiency of pA-Tn5 loading can be determined by the CUT&Tag library yield as read out on a Tapestation or Bioanalyzer.

### Sci-i5 and i7 Primer Plate:

1. Resuspend 72 barcoded i5 and 72 barcoded i7 Primers at 100 μM in 10 mM Tris pH 8 (Supplementary Table 1)^25^.
2. Prepare a master primer plate by individually pipetting 200 μL of 100 μM of barcoded i5 and i7 primers into the appropriate wells of a 384-deep well plate (Fisher Sci cat. No. 269390) and store at −20 °C for up to one year.
3. Prepare the sciCUT&Tag PCR Primer Plates by filing the appropriate wells of a 384-well ICELL8 source plate with 90 μl of 10 mM Tris pH 8 and, using a 16-channel pipette, add 10 μl of 100 μM barcoded i5 and i7 primers from the 384-deep well plate master plate to each well.
4. Using 16-channel pipette, stamp 23 μL each of the diluted 10 μM barcoded i5 and i7 primers into four iCELL8 source plates focusing on one column at a time. For sci-i5 and i7 primer plate layout see Supplementary Table 3. Store sciCUT&Tag PCR Primer Plates at 4 °C for up to one month or at −20 °C long term.

### WGA-coated MyOne-C1 magnetic beads^23^:

1. Prepare a 50 mL Stock of 1xPBS (pH 6.8) by diluting 10X PBS in Nuclease-Free Water then adjust pH by adding 1M HCl dropwise while monitoring with a pH Meter. This stock can be kept at room temperature for up to 1 year.
2. Prepare 5 mL of 1xPBS (pH 6.8) + 0.01% Tween-20 (v/v). 50 μL of 1% Tween20 (v/v) in 4950 μL of PBS (pH 6.8).
3. Resuspend 500 μl Dynabeads^™^ MyOne^™^ Streptavidin C1 in 1 mL 1xPBS (pH6.8) + 0.01% Tween-20 in standard 1.5mL Microfuge tube.
4. Place on magnet stand and allow to clear. Remove supernatant and discard without disturbing the magnetic beads. Resuspend in 1 mL 1xPBS (pH6.8) + 0.01% Tween-20 to Wash.
5. Repeat Step 3 to wash Dynabeads^™^ MyOne^™^ Streptavidin C1 a third time and then resuspend in 500 μl 1xPBS (pH6.8) + 0.01% Tween-20 in standard 1.5 mL tube.
6. Add 125 μl of 5 mg/mL Biotin-WGA to the beads with gentle vortexing to ensure even mixing.
7. Incubate on a nutator for 30 min at RT to allow Biotin-WGA to bind Dynabeads^™^ MyOne^™^ Streptavidin C1.
8. Quick spin (100xg for 2–3 seconds), and place on magnet stand for ~ 2 min to clear, then remove and discard supernatant.
9. Resuspend in 1 mL 1xPBS + 0.01% Tween.
10. Store at 4°C until use for up to six months.
  **CRITICAL:** Magnefy^™^ Concanavalin A ready-to-use beads can be used as an alternative to the WGA-coated MyOne-C1 magnetic beads, but may differ in their propensity to bind specific cell types.

**1M HEPES-KOH pH 7.9:** Add 23.83g HEPES to 80 mL of deionized water. Adjust to pH 7.0 by adding 1M KOH to solution dropwise while monitoring with a pH meter. Add deionized water to almost 100 mL then do a final pH adjustment to pH 7.9 by adding 1M KOH to solution dropwise while monitoring with a pH meter. Bring up to 100 mL with deionized water, filter sterilize and store at room temperature for up to 1 year.

**1 M HEPES pH 7.5:** Add 23.83g HEPES to 70 mL of deionized water. Adjust to pH 7.0 by adding 1M NaOH to solution dropwise while monitoring with a pH meter. Add deionized water to almost 100 mL then do a final pH adjustment to pH 7.5 by adding 1M NaOH to solution dropwise while monitoring with a pH meter. Bring up to 100 mL with deionized water, filter sterilize and store at room temperature for up to 1 year.

**NE1 buffer:** Prepare fresh by mixing 1 mL 1M HEPES-KOH pH 7.9, 500 μl 1M KCl, 12.5 μl 2 M spermidine, 500 μl 10% Triton X-100 (v/v), and 10 mL glycerol in 38 mL dH_2_O, and add 1 Roche Complete Protease Inhibitor EDTA-Free tablet. Chill on ice before use.

**Wash buffer:** Prepare fresh by mixing 1 mL 1 M HEPES pH 7.5, 1.5 mL 5 M NaCl, 12.5 μl 2 M spermidine, 250 μl of 10% (v/v) Triton-X solution and bring the final volume to 50 mL with dH_2_O, and add 1 Roche Complete Protease Inhibitor EDTA-Free tablet. Wash Buffer can be stored at 4 °C for up to 1–2 days prior to using, but a Roche Complete Protease Inhibitor EDTA-Free tablet should be added fresh before use.

**Binding buffer:** Mix 200 μl 1M HEPES-KOH pH 7.9, 100 μl 1M KCl, 10 μl 1M CaCl_2_ and 10 μl 1M MnCl_2_, and bring the final volume to 10 mL with dH2O. Store the buffer at 4 °C for up to 6 months.

**Antibody buffer:** Prepare fresh by mixing 8 μl 0.5 M EDTA with 2 mL Wash buffer and chill on ice.

**300-wash buffer:** Mix 1 mL 1 M HEPES pH 7.5, 3 mL 5 M NaCl and 12.5 μl 2 M spermidine, 250 μl of 10% (v/v) Triton-X solution, bring the final volume to 50 mL with dH_2_O and add 1 Roche Complete Protease Inhibitor EDTA-Free tablet. 300-wash buffer can be stored at 4 °C for 1–2 days prior to using, and in this case an additional Roche Complete Protease Inhibitor EDTA-Free tablet should be added fresh before use.

**Tagmentation buffer:** Prepare fresh by mixing 10 mL 300-wash buffer and 100 μl 1 M MgCl_2_ (to 10 mM).

**1 M TAPS pH 8.5 buffer:** dissolve 24.3 g TAPS ([tris(hydroxymethyl)methylamino]propanesulfonic acid) in 80 mL dH_2_O. Adjust to pH 8.5 by addition of 1 M NaOH. Bring to final 1 L volume with dH_2_O and filter sterilize.

**10 mM TAPS pH 8.5 buffer:** Mix 500 μl 1 M TAPS pH 8.5 in 50 mL dH_2_O (to 10 mM). Can be kept at Room Temperature for up to 6 months.

**0.19% SDS Release buffer:** Prepare fresh the day of the experiment by diluting 10% SDS (w/v) 1:10 in 1 mL dH_2_O. Then mix 152 μl 1% SDS and 648 μl 10 mM TAPS pH 8.5 buffer.

**2.5% Triton-X neutralization solution:** Prepare fresh the day of the experiment by mixing 250 μl 10% Triton-X100 (v/v) in 750 dH_2_0

## PROCEDURE

### *Prepare WGA-coated MyOne-C1 magnetic beads* TIMING 15 min

1. Gently resuspend and withdraw 200 μl of the WGA-coated MyOne-C1 magnetic bead slurry.
  **CRITICAL STEP:** As compared to the BioMag-Plus Concanavalin A (Bangs Laboratories, Inc.) beads used in bulk CUT&RUN and CUT&Tag protocols, the WGA-Coated MyOne-C1 beads and Magnefy^™^ Concanavalin A ready-to-use beads are smaller and more uniformly sized, reducing the formation of multi-nuclei aggregates and allowing bead-bound nuclei to pass through a 20-micron filter during split-pooling.
2. At room temperature, transfer 200 μl WGA-coated MyOne-C1 bead slurry into 1.5 mL Binding buffer in a 2 mL tube and mix by pipetting. Place the tube on a magnet stand to clear for 2 minutes.
3. Withdraw the liquid completely and remove the 2 mL tube from the magnet stand. Add 2 mL Binding buffer and mix by pipetting.
4. Place tube on magnet stand to clear, withdraw liquid, resuspend beads in 200 μl Binding buffer and leave at room temperature until nuclei are ready.

### *Prepare lightly cross-linked nuclei and cryopreserve* TIMING 1 hr

- Harvest cell culture(s) in a 15 mL conical centrifuge tube at room temperature or thaw frozen cells and count cells using an automated cell counting devise (e.g. Countess 3) or by hand using a hemocytometer. For efficient recovery of lightly cross-linked nuclei, this protocol requires starting with ~10 million mammalian cells per sample.
- Centrifuge 3 min 600 x g in a swing-bucket rotor at room temperature and drain liquid.
- Resuspend in 1.5 mL PBS at room temperature while pipetting and transfer to a 1.5 mL standard microfuge tube (not low bind) to increase visibility.
- Centrifuge 3 min 600 x g in a benchtop centrifuge at room temperature and carefully pipette off the liquid. Do not disturb the pelleted cells.
- Resuspend in 1 mL ice-cold NE1 buffer with gentle vortexing. Let sit on ice 10 min.
- Centrifuge 8 min 1300 x g at 4°C in a temperature-controlled benchtop centrifuge. Carefully pipette off the liquid. Do not disturb the pelleted nuclei.
- Resuspend in 1 mL of room temperature PBS.
- To cross-link the nuclei, add 6.4 μl of 16% formaldehyde to the sample (final concentration is 0.1%) and immediately mix by inverting the tube 5 times, then incubate at room temperature for 2 min.
- Stop cross-linking by adding 2.5 M glycine to twice the molar concentration of the formaldehyde (e.g., 30 μl to 1 mL).
- Centrifuge 8 min 1300 x g at 4°C in a temperature-controlled benchtop centrifuge. Carefully pipette off the liquid. Do not disturb the pelleted nuclei.
  **CRITICAL STEP:** Nuclei become difficult to see after cross-linking and can be quite slippery in the tube. To avoid losing nuclei, aspirate the supernatant slowly at the surface of the liquid and leave a small volume of PBS (~5 μl) at the bottom of the tube.
- Resuspend lightly cross-linked nuclei in Wash buffer and dilute to quantify nuclei using the Countess 3 or cell counter slide.
**? TROUBLESHOOTING**
- Dilute lightly cross-linked nuclei to a concentration of ~1 million nuclei/mL in Wash buffer.
  **PAUS|E POINT:** If not using immediately, nuclei may be slow frozen. To do so, aliquot 900 μl into cryogenic vials containing 100 μl DMSO, mix by pipetting, then add to a Mr. Frosty container filled to the line with isopropanol and place in a −80°C freezer overnight for storage at −80°C.

### *Bind nuclei to WGA-coated MyOne-C1 beads* TIMING 15 min

**CRITICAL**: For each sciCUT&Tag experiment, use 2 million lightly cross-linked nuclei as input. The protocol can also be run using fewer than 2 million lightly cross-linked nuclei, but this may result in suboptimal recovery. Keep freshly prepared lightly cross-linked nuclei on ice or thaw slow-frozen lightly cross-linked nuclei aliquots at room temperature. If thawing nuclei, we recommend counting nuclei prior to bead-binding on a Countess 3 instrument to account for potential lysis during freeze-thaw. For SNP-based detection of collisions and *In Silico* doublet removal during downstream analysis, it may be desirable to mix samples from multiple different donors.

- Resuspend the 200 μl of WGA-coated MyOne-C1 beads from step 4 by inverting the tube 2–3 times or by pipetting up and down with a wide-bore tip. Then, in a dropwise manner, mix 200 μl of WGA-coated MyOne-C1 beads with the nuclei suspension. To ensure even binding, close and invert samples to mix in-between additions of WGA-coated MyOne-C1 beads. Once the full 200 μl WGA-coated MyOne-C1 bead slurry has been added, close the tube and incubate on a nutator at room temperature for 10 min.
  **CRITICAL STEP:** If numerous distinct samples are to be profiled in the same experiment, the proportion of nuclei and the WGA-coated MyOne-C1 bead slurry should correspond to the proportion of the 96-well sci-pA-Tn5 plate that will be dedicated to each sample (for example, for an experiment with a total of 2 million nuclei split evenly between K562 cells, H1 cells and PBMCs, ~670K nuclei of each cell type should be bound to 67 μl WGA-coated MyOne-C1 beads).
- Divide the sample into 0.65 mL pre-lubricated microcentrifuge tubes at the appropriate ratio for each antibody to be profiled.
  **CRITICAL STEP**: The number of nuclei per antibody can vary greatly depending on the experimental design (For example, for an experiment in which two different histone modifications will be profiled in an equal number of nuclei, the sample should be split evenly between two 0.65 mL pre-lubricated microcentrifuge tubes. In an experiment in which four different histone marks will be profiled and two of the histone marks will be profiled in twice as many cells as the other two histone marks, the sample will be split into four tubes: 1/3 the volume of bead-bound nuclei will be allocated to the two “high-cell-number histone marks” and 1/6 the volume of bead-bound nuclei being allocated to the two “low-cell-number histone marks”. Each of the four samples will then be allocated a corresponding proportion of the 96-well plate for sci-pA-Tn5 binding and tagmentation).
- Place the tubes on a magnet stand to clear and withdraw the liquid. If desired, the unbound supernatants may be counted on a Countess 3 instrument to check efficiency of nuclei binding to beads.
**? TROUBLESHOOTING**

### ***Bind primary antibody*** TIMING 1 hr 15 min

- Resuspend samples in Antibody Buffer at a concentration of ≤ 20,000 bead bound nuclei per μl (for example, bring up 1 million bead bound nuclei to 50 μl in Antibody Buffer). Then add the relevant antibodies to each sample at a 1:10 dilution (v/v) of Antibody solution to sample (for example, add 5 μL of anti-H3K27me3 antibody to 50 μL of sample). If multiple antibodies are to be added to a single sample (e.g., anti-H3K4me1, anti-H3K4me2 and anti-H3K4me3 antibodies), the volume of each antibody solution should correspond to 1/10 the initial volume of the sample.
- Mix by gentle vortexing, or flicking the tube, then quick spin (100xg for 2–3 seconds) to collect the solution at the bottom of the tube.
- Place in a tube rack at room temperature and incubate for 30 minutes, then mix by gentle vortexing or flicking the tube, quick spin to collect solution at the bottom of the tube (100xg for 2–3 seconds) and incubate for an additional 30 minutes at room temperature or at 4˚C overnight.
  **PAUSE POINT** Antibody incubation may proceed overnight at 4 °C; we have not noticed any difference between the efficiency of a 1 hr room temperature incubation and an overnight 4 °C incubation.

### ***Bind secondary antibody*** TIMING 45 min

- Quick spin at 100xg for 2–3 seconds, place each tube on the magnet stand to clear for 2 min and remove the liquid from each tube by pipetting.
  **CRITICAL:** The quick spin on a micro-centrifuge minimizes the carry-over of reagents that could result in increased background tagmentation or an overall reduction in pA-Tn5 tethering to targeted chromatin.
- Remove tubes from the magnet stand and wash each with 150 μl of Wash buffer. Place each tube back on the magnet stand to clear for 2 minutes and remove the liquid.
- Repeat step 24 for a second wash.
- Resuspend samples in 30 μl of Wash buffer and add 1.5 μl secondary antibody (1:20). Mix by gentle vortexing, or flicking the tubes, then quick spin (100xg for 2–3 seconds) to collect the solution at the bottom of the tube.
- Place the tubes in a tube rack at room temperature for 30 min or 4 °C for 1 hr. Halfway through the incubation, again mix by gentle vortexing, or flicking the tube, then quick spin to collect the solution at the bottom of the tube.
  **PAUSE POINT** Secondary antibody incubation may proceed overnight at 4 °C.
- After a quick spin (100xg for 2–3 seconds), place each tube on the magnet stand to clear for 2 min and remove the liquid from each tube by pipetting.
- Wash with 150 μl of Wash buffer. Place each tube on the magnet stand to clear for 2 min and remove the liquid from each tube by pipetting.
- Repeat step 29 for a second wash.

### *Bind pA-Tn5 adapter complex* TIMING 1 hr 15 min

- Resuspend WGA-coated MyOne-C1 bead bound samples in 300-wash buffer to a final volume of 20 μl per well of the 96-well sci-pA-Tn5 plate (for example, if the experiment is split evenly between two samples, 48 wells each, then both samples should be brought up in 960 μl 300-wash buffer. Include an additional 20 μl of 300-wash buffer to avoid running out of solution while loading the plate).
- Transfer 20 μL of the appropriate sample into each well of a fresh 96 well LoBind Semi-skirted PCR plate.
  **CRITICAL STEP:** Be sure to track the distribution of each sample across the plate for demultiplexing after sequencing.
- Centrifuge the sealed Sci-pA-Tn5 Plate for 1 minute at 1200 g before opening.
- Using an 8-channel 20 μl multi pipette, transfer 2 μl of adapter-loaded pA-Tn5 from the Sci-pA-Tn5 Plate into the corresponding wells of the 96 well sample plate. Mix by pipetting up and down ~5 times. Seal the Sci-pA-Tn5 Plate and store at −20 °C.
- Allow pA-Tn5 binding to proceed for 1hr at 4 °C while keeping the 96 well sample plate stationary.
- Add 50 μl of 300-wash buffer to each well of the 96 well sample plate. Then place on the 96 well plate ring magnet. Allow samples to collect on the side of the wells (~1 min).
- Using an 8-channel 200 μl multi pipette, remove unbound pA-Tn5 solution and discard in waste.
  **CRITICAL STEP:** During wash steps, avoid cross-contamination of pA-Tn5 solution between wells.
- Decant 300-wash buffer into a reservoir and add 150 μl to each well of the 96 well sample plate. Incubate for 2–5 min while keeping the sample plate on the magnet.
- Using an 8-channel 200 μl multi pipette, remove wash solution and transfer to waste.
- Repeat steps 38–39 (second wash).

### *Tagmentation* TIMING 1 hr 15 min

- Decant Tagmentation buffer into a reservoir and add 75 μL to each well of the 96 well sample plate.
- Seal sample plate with an AlumaSeal 96 cover, and place in a ThermoCycler set to 37°C with heated lid for 1 hr.

### *Repool, Filter and Count* TIMING 40 min

- Place 96 well sample plate on the 96 well plate ring magnet. Allow samples to collect on the side of the wells (~1 min). Remove AlumaSeal and using an 8-channel 200 μl multi pipette, remove Tagmentation Buffer and discard in waste.
- Remove the 96 well sample plate from the magnet. Decant Wash buffer into a reservoir and using an 8-channel 200 μl multi pipette with wide-bore tips transfer 200 μl Wash buffer into the 8 wells of column 1 of the sample plate.
- Gently pipette the Wash buffer up and down, dispensing the solution along the side of the well in a circular motion to dislodge all the bead-bound nuclei from the sides of the wells and resuspend them in solution.
- Transfer the 200 μl Wash buffer with bead-bound tagmented nuclei from column 1 into the 8 wells of column 2.
- Repeat Step 45 to resuspend the bead-bound nuclei from column 2.
- Transfer the 200 μl Wash buffer with bead-bound tagmented nuclei from columns 2 and 3 into the 8 wells of column 3. Repeat Steps 45 and 46 for each successive column to collect all the nuclei from each row in 200 μl of Wash buffer in column 12.
- Using a single-channel 200 μl pipette with wide-bore tips, transfer Wash buffer and nuclei from each well of column 12 to a 0.65 mL pre-lubricated microcentrifuge tube on a magnet stand. As the tube fills up, wait for the beads to clear for 2 min, and transfer clear Wash buffer to the waste.
- Repeat Step 49 until all the bead-bound nuclei have been collected from the 96 well sample plate into the 0.65 mL pre-lubricated microcentrifuge tube.
- Remove tube from the magnet stand and wash bead-bound nuclei with 500 μl Wash buffer, pH 8.5.
- Return sample to the magnet stand and allow to clear for 2 min, then transfer Wash buffer to waste.
- Remove sample from the magnet stand and resuspend bead-bound nuclei in 540 μl Wash buffer.
- Place two 20 μm Mini-Strainers in two open 1.7-mL Low Retention Microtubes.
- Using a single-channel 200 μl pipette with wide-bore tips, transfer 270 μl of sample to each of the 20 μm Mini-Strainers
- Carefully move the loaded 20 μm Mini-Strainers and Low Retention Microtubes to a bench-top centrifuge.
- Centrifuge for 3 min at 100 g at room temperature.
  **CRITICAL STEP:** Passing the sample solution through a 20 μm Mini-Strainer is necessary to remove any large multi-nuclear aggregates prior to loading the ICELL8. Following centrifugation, the surface of the 20 μm Mini-Strainer should appear brown from the visible WGA-coated beads that are filtered out along with the multicellular aggregates.
- If liquid remains on top of the 20 μm Mini-Strainers, twist the strainer to break any seal with the lip of the Low Retention Microtubes and repeat Step 57.
- Using a single channel 200 μl pipette with wide-bore tips, recombine the two filtered samples into a single 1.7-mL Low Retention Microtube.
- Dilute a 10,000X DAPI stock 1:100 in 10 mM TAPS buffer pH 8.5, and then add 5.4 μl of the 100X DAPI solution to the sample solution for a final concentration of 0.5 μg/mL DAPI.
  **CRITICAL STEP:** DAPI is used for detecting and counting nuclei on the Countess Cell Counter as well as the ICELL8. To obtain an accurate count of the concentration of nuclei, DAPI staining is necessary to distinguish bead-bound nuclei from free-floating beads.
- Load 10 μl of Sample solution onto a plastic slide for the Countess.
- Insert the slide into the Countess then turn on the bright field and DAPI Channels and image for quantification.
  **CRITICAL STEP:** To obtain an accurate quantification of DAPI-positive nuclei, it may be necessary to (1) adjust the intensity of the DAPI laser or (2) adjust the detection parameters for calling nuclei during the image processing. Typically, an accurate count ranges from 1 × 10^5^ DAPI-positive nuclei/mL to 4 ×10^5^ DAPI-positive nuclei/mL.
- Repeat Steps 61 and 62 three more times to get four replicate counts of the DAPI-positive nuclei/mL.
**? TROUBLESHOOTING**

### *Dispense on the ICELL8, Image and Perform SDS Release.* TIMING 2 hr 30 min

- Calculate the average of the four replicate counts of the DAPI-positive nuclei/mL.
- Thaw one aliquot of the 100X Diluent and the Fiducial reagents from the SMARTer ICELL8 Blank Chip Reagent Kit at room temperature.
- Prepare the ICELL8 by running (1) a system prime and (2) a daily warmup. Turn on the microscope system for imaging the chip.
- Place a 350v chip on the target platform within the humidity-controlled chamber of the ICELL8.
- Load 25 μl of 10 mM TAPS pH 8.5 into the positive and negative control wells of a 384 well ICELL8 loading plate (A24 and P24).
- Load 25 μl of the fiducial reagent into the fiducial well of the 384 well ICELL8 loading plate (P1).
- Place sample on a magnet stand and allow to clear for 2 minutes. Discard Wash Buffer plus DAPI and resuspend the bead-bound sample in 10 mM TAPS pH 8.5 at a concentration of 2.85 – 5.71 × 10^5^ DAPI-positive nuclei. Then add the appropriate volume of 100X DAPI solution and the 100X Diluent so that the final concentration in the sample is 1X DAPI and 1X Diluent. Proceed immediately to sample dispense.
  **CRITICAL STEP:** The minimum volume necessary to load the ICELL8 is 540 μL and the maximum concentration of nuclei that should be dispensed on the ICELL8 is 5.71 × 10^5^ DAPI-positive nuclei/mL, which corresponds to 20 nuclei per 35 nL dispense. If a sufficient number of tagmented nuclei are obtained, it may be desirable to increase the volume of the sample to 800 μL which is enough to dispense two 350v ICELL8 chips; this strategy captures more single cells, but also requires an additional flow cell for sequencing.
**? TROUBLESHOOTING**
- Using a wide bore 200 μl tip, homogenize the sample by pipetting up and down 3–4 times then load 65 μl of sample solution into each of the 8 source wells for cellular dispense on the 384 well ICELL8 loading plate (rows A-D, columns 1&2).
  **CRITICAL STEP:** During protocol optimization, it may be desirable to perform a serial dilution experiment. For example, we performed a serial dilution to determine the collision frequency at different loading concentrations. For this, load different concentrations of sample into the 8 wells of the source plate.
- Secure the source plate in the humidified chamber of the ICELL8
- Make sure to load the Pause and Dispense protocol prior to dispensing cells. Proceed with an unfiltered, 35 nL cell dispense cycle. When prompted, mix the sample in the source wells prior to each dispense by pipetting up and down 5 times using a wide bore tip, and then resume dispensing.
- Once the cell dispense is complete, remove the 350v chip from the humidity-controlled chamber of the ICELL8. Dry the 350v chip with the circular paper from the SMARTer ICELL8 Loading Kit, then cover the chip with the translucent three leaf imaging film provided in the SMARTer kit and seal.
- Using the ICELL8 chip adapter and balance provided with the ICELL8, spin at 1200 x g for 1 min at room temp.
- Image the ICELL8 chip to examine the distribution of nuclei at different regions of the plate. Here, we would like to obtain 10–20 nuclei per well, and the imaging is used only as a quality control to examine the distribution of nuclei across the chip.
  **CRITICAL STEP:** This combinatorial indexing approach does not require identifying wells that contain a single nucleus, or the generation of a filter file for downstream dispenses.
**? TROUBLESHOOTING**
- Remove the imaging film and place the 350v chip on the target platform within the humidity-controlled chamber of the ICELL8.
- Load a fresh 384 well plate with the 0.19% SDS Release buffer. Dispense 25 μl of the 0.19% SDS Release buffer into the positive control, negative control and fiducial wells (A24, P24 and P1). Dispense 80 μl of the 0.19% SDS Release buffer into the 8 source wells for cellular dispense (rows A-D, columns 1&2).
  **CRITICAL STEP:** During the 58 °C Tn5-release step, the concentration of SDS is 0.095% (w/v). This concentration has been optimized to maximize Tn5 release while allowing for consistent quenching of the SDS with Triton-X prior to the addition of PCR reagents^30^.
- Secure the source plate in the humidified chamber of the ICELL8.
- Proceed with an unfiltered, 35 nL cell dispense cycle.
  **CRITICAL STEP:** Because all other solutions remain homogenous throughout dispense cycles, the rest of the protocol does not require the Pause and Dispense protocol.
- Once the SDS Release buffer dispense is complete, remove the 350v chip from the humidity-controlled chamber of the ICELL8. Dry the 350v chip with the circular paper from the SMARTer ICELL8 Loading Kit, then cover with the thermal film (white backing) and seal.
- Using the ICELL8 chip adapter and balance, spin at 1200 x g for 1 min at Room Temperature.
- Place the 350v chip in a thermocycler modified with the ICELL8 chip adapter. Incubate for 1hr at 58°C, then hold at 12°C.
  **PAUSE POINT:** Following the 1hr incubation at 58°C the chip can be wrapped in parafilm and stored at 4°C indefinitely.

### *Triton-X Quench and PCR amplification.* TIMING 1 hr 30 min

- Using the ICELL8 chip adapter and balance, spin the sealed 350v chip at 1200 x g for 1 min at room temp.
- Remove the thermal film and place the 350v chip on the target platform within the humidity-controlled chamber of the ICELL8.
- Load 80 μl of the 2.5% Triton-X Neutralizing solution into the 8 source wells of the 384 well sciCUT&Tag PCR Primer Plate (rows A-D, columns 1&2). Add 25 μL 10 mM TAPS to fiducial, negative and positive control wells (P1, A24, P24).
- Secure the sciCUT&Tag PCR Primer source plate in the humidified chamber of the ICELL8.
- Proceed with an unfiltered, 35 nL cell dispense cycle.
  **CRITICAL STEP:** Similar to the 0.19% SDS Release buffer dispense, here, the cell dispense cycle is used for dispensing 35 nL of the Triton-X Neutralizing solution into all the wells of the 350v chip.
- Once the dispense is complete, remove the 350v chip from the humidity-controlled chamber of the ICELL8. Dry the 350v chip with the circular paper from the SMARTer ICELL8 Loading Kit, then cover with either an imaging film or thermal film and seal.
- Using the ICELL8 chip adapter and balance, spin at 1200 x g for 1 min at room temp.
- Remove the film and return the 350v chip to the target platform within the humidity-controlled chamber of the ICELL8.
- Proceed with the 35 nL unfiltered dispense of the first set of sciCUT&Tag primers (Index1, sci i5s).
- Repeat Steps 89–91 to dry and spin down the contents of the chip between dispenses.
- Proceed with the 35 nL unfiltered dispense of the second set of sciCUT&Tag primers (Index2, sci i7s).
- Repeat Steps 89–91 to dry and spin down the contents of the chip between dispenses.
- Prepare the KAPA HiFi PCR Mix. In a 1.5-mL microcentrifuge tube, mix 553.7 μl 5X HiFi Buffer, 306.6 μl ddH_2_O, 84.8 μl dNTPs (10mM) and 54.9 μl KAPA HiFi Polymerase.
- Load 100 μl of KAPA HiFi PCR Mix into the 8 source wells for cellular dispense on the 384 well ICELL8 loading plate (rows A-D, columns 1&2) and save remaining PCR mix. Add 25 μL 10 mM TAPS to fiducial, negative and positive control wells (P1, A24, P24).
- Secure the source plate in the humidified chamber of the ICELL8.
- Proceed with a 50 nL unfiltered cell dispense of the KAPA HiFi PCR Mix.
- Repeat Steps 89–91 to dry and spin down the contents of the chip between dispenses.
- Load an additional 24 μl of KAPA HiFi PCR Mix into the 8 source wells for cellular dispense on the 384 well ICELL8 loading plate (rows A-D, columns 1&2).
- Secure the source plate in the humidified chamber of the ICELL8.
- Proceed with a second 50 nL unfiltered cell dispense of the KAPA HiFi PCR Mix.
- Once the dispense is complete, remove the 350v chip from the humidity-controlled chamber of the ICELL8. Dry the 350v chip with the circular paper from the SMARTer ICELL8 Loading Kit. Then cover with a thermal film and seal well to avoid evaporation.
- Using the ICELL8 chip adapter and balance, spin at 1200 x g for 1 min at room temp.
- Place the 350v chip in a thermocycler modified with the ICELL8 chip adapter.
- Proceed with PCR reaction according to the following PCR cycling conditions).

**Table:** 

Cycle number	Denature	Anneal	Extend	Final
1			58° C, 5 min	
2			72° C, 10 min	
3	98° C, 45 s			
4–19	98° C, 15 s	60° C, 10 s	72° C, 5 s	
20			72° C, 1 min	
21				8° C, hold

### *Sample Collection* TIMING 15 min

- Open the ICELL8 Collection Kit and assemble the collection module by attaching the 0.7 mL microfuge tube to the chip collection funnel. Remove the thermal film from the 350v chip and invert the chip, with the nanowells facing down, onto the chip collection funnel. Insert the collection module into the ICELL8 collection adapter. Seal the chip to the collection module with the provided sealing tape.
- Using the ICELL8 collection adapter balance, centrifuge the collection assembly at 1200 x g for 8 sec at room temp and then stop. Transfer the sample to a 1.7 mL tube, re-assemble the collection module, repeat the 8 sec centrifugation step, and transfer the sample. Centrifuge the chip for 5 min and collect the remaining sample.
  **CRITICAL STEP:** The volume of PCR product exceeds the capacity of a single 0.7 mL microfuge collection tube. To avoid overflow, the sample must be collected in intervals.

### *Post-PCR Cleanup* TIMING 1 hr 15 min

- Determine the volume of the PCR amplified sciCUT&Tag library by adjusting the volume of a 1 mL Pipette until it matches the sample volume upon aspiration. Split evenly between two 1.7 mL low retention tubes and add 1.1 x the volume of Ampure XP beads, mixing by pipetting up and down.
- Quick spin (100xg for 1–2 seconds) and let sit at room temperature 5–10 min.
- Place the tubes on a magnet stand and allow the solutions to clear for 3 min before carefully withdrawing liquid. While the empty tubes are still on the magnet stand, add 1.5 mL 80% ethanol to each tube.
- Allow solutions to clear before carefully withdrawing liquid. While still on the magnet, add 1.5 mL 80% ethanol.
- Remove liquid and discard.
  **CRITICAL STEP:** Overdrying can reduce recovery of DNA, so continue to the elution step within 5 min.
- Remove from the magnet stand, add 100 μL 10 mM Tris-HCl pH 8 to each of the tubes and vortex on full for 30 seconds. Then collect sample at the bottom of the tube using a quick spin (100xg for 2–3 sec).
- Incubate tubes for 5 min at room temperature and then place the tubes on the magnet stand and allow to clear for 2 min.
- Combine the two 100 μL 10 mM Tris-HCl pH 8 solutions containing the eluted sciCUT&Tag library into a single fresh 1.5 mL tube with a pipette.
- Add 0.9 x the volume of Ampure XP beads (180 μl), mixing by pipetting up and down.
- Quick spin (100xg for 2–3 seconds) and let sit at room temperature 5–10 min.
- Place tube on the magnet stand and allow to clear before carefully withdrawing liquid. While the tube is still on the magnet, add 500 μl 80% ethanol.
- Allow to clear before carefully withdrawing liquid. While the tube is still on the magnet stand, add 500 μl 80% ethanol.
- Withdraw the liquid and discard in liquid waste.
  **CRITICAL STEP:** Over-drying can reduce recovery of DNA, so continue to the elution step within < 5 min.
- Remove from magnet stand, add 25 μl 10 mM Tris-HCl pH 8 and vortex on full for 30 seconds.
- After a 5 minute incubation at room temperature place on the magnet stand and allow to clear for 2 minutes.
- Transfer the 25 μl 10 mM Tris-HCl pH 8 solution containing the eluted sciCUT&Tag library to a fresh 1.5 mL tube with a pipette.
  **PAUSE POINT:** Libraries can be stored at 4˚C for up to two weeks or at −20˚C for up to 1 year.

### *Library analysis and DNA sequencing* TIMING: 1 d

- Determine the size distribution of libraries by capillary electrophoresis (*e.g.* using the Agilent 4200 TapeStation) using 2 μl of the library, following the manufacturer’s instructions (https://www.agilent.com/cs/library/usermanuals/public/4200-TapeStation_SystemManual.pdf). For mixing single-tube samples with load buffer, use a low-volume (*e.g.* 2 μl) pipettor to avoid injecting bubbles.
  **CRITICAL STEP:** Molarity estimates are based on the capillary electrophoresis profile by choosing the region between 160 bp and 1000 bp and using the value reported as “Region Molarity [pmol/L]”.
**? TROUBLESHOOTING**
- Allow the Illumina NextSeq 2000 Flow Cell and cartridge to thaw at room temperature for 15 minutes.
  **CRITICAL STEP:** The Next-Seq P2–200 is expected to produce 400 Million reads and is preferable to obtain sufficient library saturation for samples with an initial concentration > 10 nM. The Next-Seq P1–300 is expected to produce 100 Million reads and is preferable to reduce the cost while obtaining sufficient library saturation for samples with an initial concentration < 10 nM.
- Remove cartridge from the bag and invert it 10 times to mix reagents.
- For sequencing on a NextSeq2000 instrument, dilute the sciCUT&Tag library to 2nM using the RSB with Tween supplied as part of the Illumina flow cell kit. Then dilute to the final loading concentration specified in the Illumina Onboard Denature and Dilute Guide. We do not include the PhIX spike in.
- Resuspend NextSeq_Read1_seq_primer, NextSeq_Read2_seq_primer, NextSeq_Index1_seq_primer and NextSeq_Index2_seq_primer at 100 μM in 10 mM Tris HCl pH 8.0 (For sequences see Supplementary Table 1).
- For loading, mix 1.8 μL of NextSeq_Read1_seq_primer and 1.8 μL of NextSeq_Read2_seq_primer and dilute to 0.3 μM, by adding 596.4 μL HT1 Buffer.
- Mix 3.6 μL of NextSeq_Index1_seq_primer and 3.6 μL of NextSeq_Index2_seq_primer and dilute to 0.6 μM, by adding 592.8 μL HT1 Buffer.
- Using a new P1000 tip, pierce the Library Reservoir and push the foil to the edges.
- Add 20 μL of the diluted sciCUT&Tag library to the bottom of the Library Reservoir. Avoid touching the foil.
- Mix the 0.3 μM Read1&2 primer solution by pipetting up and down 4 times and then transfer 550 μL to the well “Custom 1” on the Illumina instrument.
- Mix the 0.6 μM Index1&2 primer solution by pipetting up and down 4 times and then transfer 550 μL to the well “Custom 2” on the Illumina instrument.
- Pull the flow cell out of the package and hold by the gray tab with the label facing up.
- Push to insert the flow cell into the slot on the front of the cartridge.
- Pull back the gray tab and remove to expose the flow cell.
- Click Load to load the cartridge.
- Wait 2–3 minutes while the sequencer is scanning consumables. Then double check the Sequencing configuration and press Sequence.

### *Data Processing and Analysis* TIMING: Variable compute timing

- Perform demultiplexing using the sciCTextract custom software. This requires six input files: the four Fastq files corresponding to genomic read 1, genomic read 2, index read 1 and index read 2, as well as two barcode tables in comma-separated value format (.csv). The Fastq files should follow Illumina naming conventions, including read headers (e.g. @VH00319:342:AACKYJMM5:1:1101:31410:1000 1:N:0:0). The first barcode.csv file defines the prefixes of the sample names based on the s5 and s7 adapter sequences (Supplementary Table 4). The second barcode.csv file defines the suffixes of the sample names based on the i5 and i7 primer sequences (Supplementary Table 5). The output of sciCTextract consists of a pair of compressed Fastq files with rewritten headers that include the error corrected barcode sequences, as well as enough information to unambiguously identify each source read (e.g. @AACKYJMM5:1:1101:31410:1000_GCGTTAAA_GTGTATCG_AGCGATAG_CAGGACGT 1:N:0:0).
- Clip adapter sequences using cutadapt 2.9 with parameters: -j 8 -m 20 -a CTGTCTCTTATACACATCT -A CTGTCTCTTATACACATCT -Z
- Perform alignments using Bowtie2 version 2.4.2 to UCSC HG38 with parameters: --very-sensitive-local --soft-clipped-unmapped-tlen --no-mixed --no-discordant --dovetail --phred33 -I 10 -X 1000
- Convert the sam file created by bowtie2 to a bam file and run “bedtools bamtobed -bedpe” on the resulting bam file.
  **CRITICAL STEP:** If the experiment includes multiple donor samples mixed together, perform SNP-based identification of doublets using the aligned reads as input to Souporcell^24^. To streamline this process, we assembled an easy-to-use Nextflow pipeline called Souper-star that removes duplicate reads, merges files for Souporcell, generates quality control output, and splits out bed files for each epitope profiled (https://github.com/FredHutch/souper-star).
- From the bed file, extract columns 1, 2, 6, 7 which correspond to the chr, start, end and the rewritten headers including the barcode.
- Prepare the bedfile for input into single-cell analysis software such as ArchR by reformatting the fourth column to include just the barcode sequence and removing duplicate reads with the same chromosome, start, stop, and barcode. This can be done using the bash script: cat $1 | awk ‘{gsub(“:”,”\t”,$4);gsub(“_”,”\t”,$4); print $1”\t”$2”\t”$3”\t”$4”\t”$5}’ | awk ‘{print $1”\t”$2”\t”$3”\t”$9”_”$10”_”$11”_”$12}’ | sort -k1,1 -k2,2n -k4,4 | uniq -c | awk ‘{print $2”\t”$3”\t”$4”\t”$5”\t”$1}’ > $2
- Use the resulting bed file as the input to a single-cell analysis package for dimensionality reduction and graph-based clustering (*e.g*., using ArchR, Signac, chromVAR^31^). Aggregate bed files can also be used for peak-calling (SEACR^32^). Example analysis can be found here: https://github.com/FredHutch/sciCnT_manuscript_2023.

## TIMING

Steps 1–4, Prepare WGA-coated MyOne-C1 magnetic beads: 15 min

Steps 5–16, Prepare lightly cross-linked nuclei and cryopreserve: 1 hr

Steps 17–19, Bind nuclei to WGA-coated MyOne-C1 beads: 15 min

Steps 20–22, Bind primary antibody: 1 hr 15 min

Steps 23–30, Bind secondary antibody: 45 min

Steps 31–40, Bind pA-Tn5 adapter complex: 1 hr 15 min

Steps 41–42, Tagmentation: 1 hr 15 min

Steps 43–63, Repool, filter and count: 40 min

Steps 64–83, Dispense on the ICELL8, image and perform SDS Release: 2 hr 30 min

Steps 84–107, Triton-X Quench and PCR amplification: 1 hr 30 min

Steps 108–109, Sample collection: 15 min

Steps 110–125, Post-PCR cleanup: 1 hr 15 min

Steps 126–141, Library analysis and DNA sequencing: 1 day

Steps 142–148, Data processing and analysis: variable

### Troubleshooting

Troubleshooting advice can be found in Table 2.

### Anticipated results

#### Sequencing results and distribution of reads across barcodes

We demonstrated the sciCUT&Tag protocol by mixing human PBMCs from two donors and profiling H3K27me3 and H3K4me1–2-3. H3K27me3 labels regions of Polycomb-mediated developmental gene repression. Of note, we pooled antibodies to target all methylated forms of H3K4, which yielded biologically interpretable signal as H3K4 methylation is controlled by highly conserved enzymatic complexes that label nucleosomes at regions of transcriptionally active chromatin (i.e., active promoters and enhancers)^33^.

**Fig. 4:**
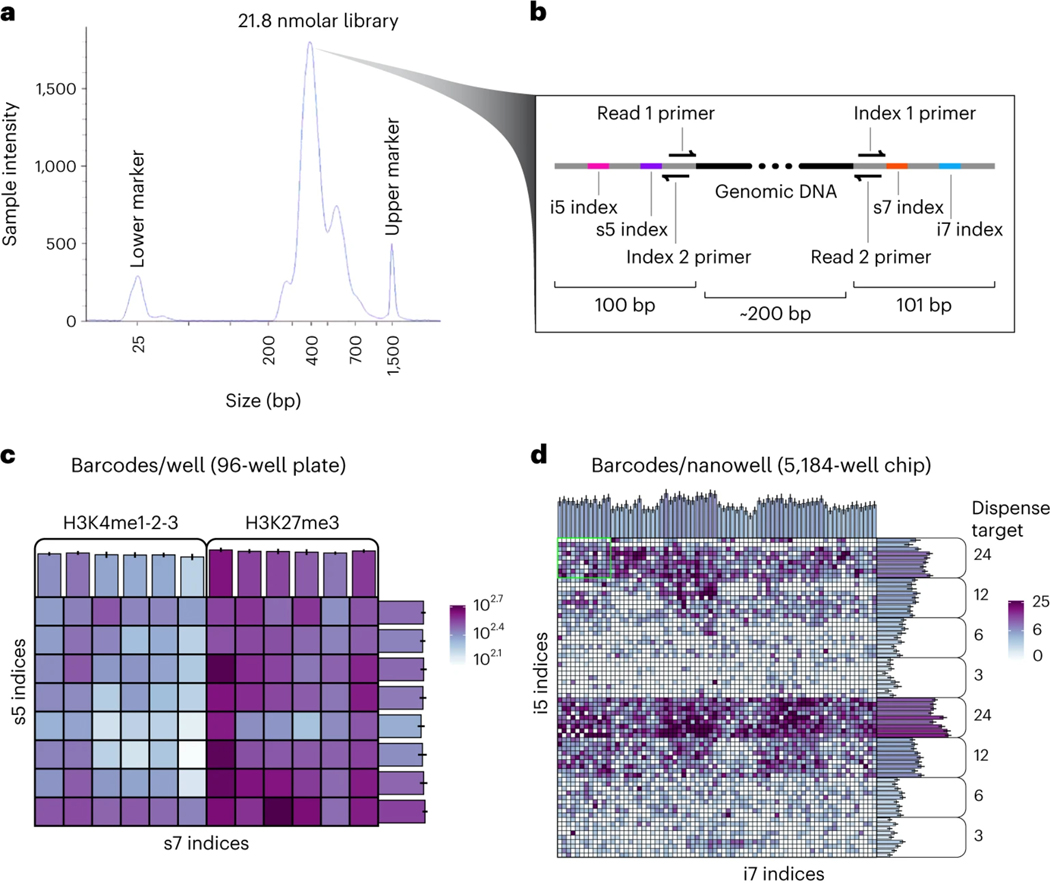
Tunable combinatorial indexing in sciCUT&Tag with the ICELL8 system. a, Readout from the TapeStation for sciCUT&Tag profiling of H3K27me3 and H3K4mel-2–3 in PBMCs showing fragment size distribution, b, sciCUT&Tag barcoding schema, c, A 96-well plate heat map shaded per well by unique barcode combinations with greater than 100 reads recovered by sequencing. Marginal bar plots show the mean number of unique combinations recovered per s5 and s7 index and are shaded accordingly. Marginal bar plots for the s7 indices are labeled by profiled epitope. Error bars are standard deviation, d, 5,184-well chip shaded by unique barcode combinations with greater than 100 reads recovered by sequencing per well. Marginal bar plots show the mean number of unique combinations recovered per column and row and are shaded accordingly. Marginal bar plots along thê-axis are labeled by the cell/well dispense target. Error bars are standard error.

The results of the quality control step described in step 126 are shown in Fig. 4. The undiluted library that contains barcoded reads for both H3K27me3 and H3K4me1–2-3 profiles yielded a peak at around 400 bp (Fig. 4a), which is anticipated given the sciCUT&Tag barcoding schema (Fig. 4b). Paired-end sequencing at 79 X 43 X 37 X 79 cycles was then be performed with a Next-Seq P2–200 kit.

For preliminary analysis of demultiplexed reads described in step 142, we first calculated the number of unique barcode combinations sequenced and map them to the 96-well plate using the s5 and s7 indices. These indices distinguish the H3K27me3 and H3K4me1–2-3 wells in the same 96-well plate (Fig. 4b), and we recovered 363 barcodes/well for H3K27me3 (*%yield = 1.7; stdev. = 73.8, n = 48* ) and 240 barcodes/well for H3K4me1–2-3 (*%yield = 1.2; stdev. = 57.4, n = 48*). There should be little variation in mean recovery across the eight s5 and twelve s7 indices, which was observed (Fig. 4c and marginal bar plots). Also, the relatively low percent recovery is expected and results from (1) passing the sample through a 20 μm Mini-Strainer to remove multicellular aggregates and (2) loading only a fraction of the sample to obtain between 3–24 nuclei / nanowell on the ICELL8 depending on the experiment (see below).

During library preparation, tagmented nuclei from the 96-well plate are pooled and then dispensed in the 5184-well chip in blocks of 96 that include control wells (Fig. 4c, green rectangle). We then performed a similar analysis described in step 142–145 to map the number of unique barcode combinations sequenced to the 5184-well chip using the i5 and i7 indices. A key metric that affects the rate of collisions is the number of cells/nanowell, so we programmed the ICELL8 to perform a serial dilution in duplicate at 24, 12, 6, and 3 cells/nanowell (Fig. 4d). Concordantly, the serial dilution was reflected in mean recovery per nanowell, as indicated by the stepwise reduction in barcodes/nanowell along the y axis of the chip (Fig. 4d). Combinatorial indexing with the ICELL8 system therefore affords sciCUT&Tag a high degree of tunable cell-per-well accuracy.

#### Measuring signal-to-noise and antibody specificity with genomic coverage and peak calling analysis

An important step in data validation for chromatin profiling is measuring signal-to-noise to confirm that profiling is targeted. The signal for antibodies that target distinct epitopes should be likewise distinct, otherwise the given antibodies are non-specific. For chromatin profiling experiments, signal-to-noise can be visually inspected with tools like integrated genomics viewer (IGV)^34^ or the UCSC genome browser^35^, as well as with peak-calling algorithms that distinguish enrichment in genomic coverage over background. While peak-calling algorithms have yet to be developed for single-cell chromatin profiling data, it is possible to apply bulk peak-calling algorithms to aggregated single-cell data, and we recommend using algorithms for sciCUT&Tag that are designed for bulk CUT&RUN and CUT&Tag data, such as SEACR^32^.

**Fig. 5:**
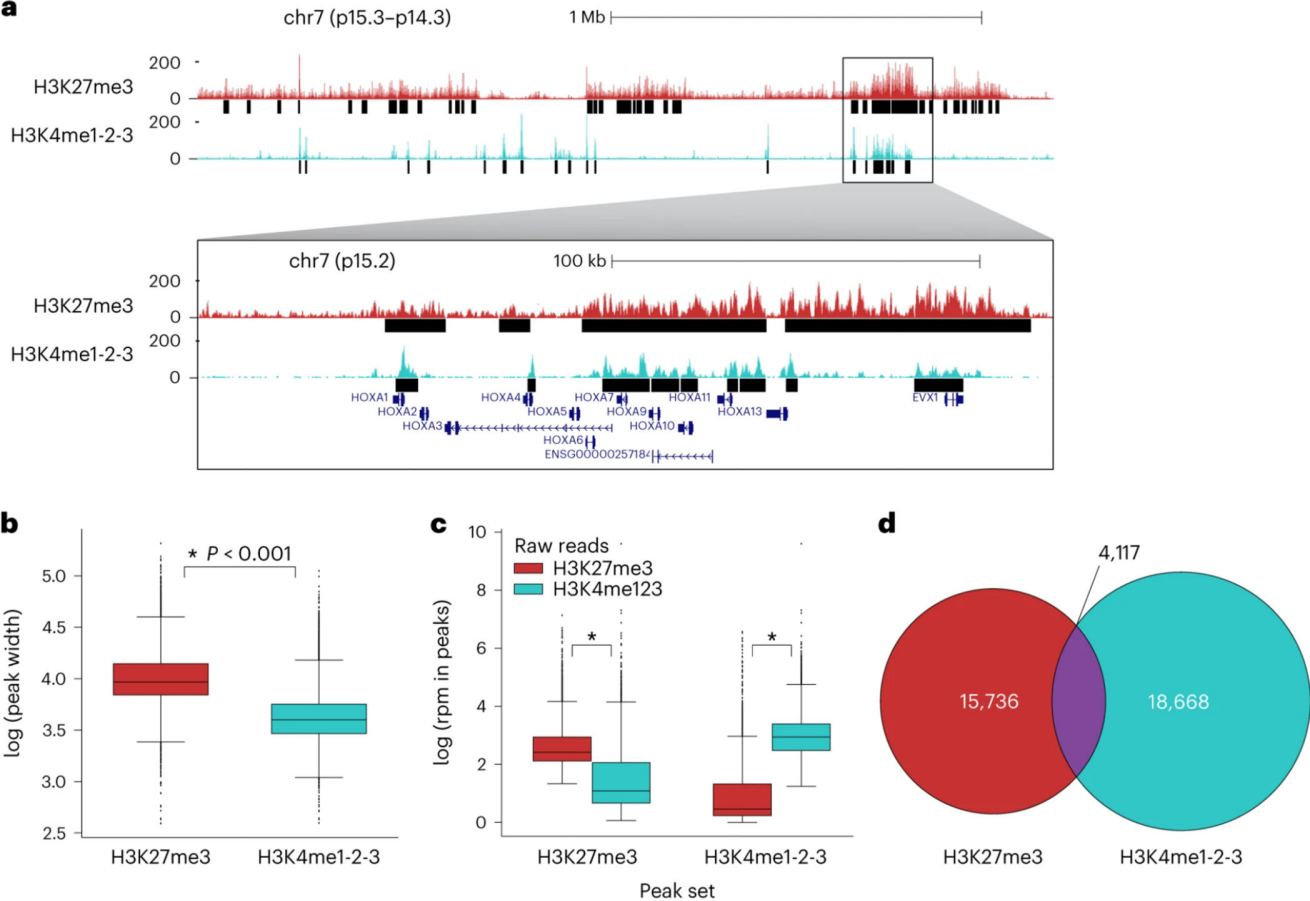
Visual inspection of genomic coverage and peak-calling analysis. a, Genomic coverage at the HOXA locus on chromosome 7 for H3K27me3 (red) and H3K4mel-2–3 (teal) aggregated profiles. Peaks detected by SEACR^ (black) are plotted below their corresponding track, b, Peak width comparison between H3K27me3 and H3K4mel-2–3 peak sets (independent f-test). Box plots show the median with first and third quartiles and outliers, c, Comparison of H3K27me3 and H3K4mel-2–3 reads per million in the two respective peak sets. Raw reads per peak were normalized by total fragment counts per million. Box plots show the median with first and third quartiles and outliers, d, Quantification of overlapping peaks across the H3K27me3 and H3K4mel-2–3 peak sets.

For example, we used SEACR to call enriched regions in aggregated sciCUT&Tag profiling of H3K27me3 and H3K4me1–2-3 in PBMCs by selecting the top 1% of regions by area under curve (AUC). Peaks can be plotted with genomic coverage and visualized in the UCSC genome browser^35^. We observed that genomic coverage at the *HOXA* locus, which is differentially regulated during hematopoiesis^36^, varied substantially between H3K27me3 and H3K4me1–2-3 bulk profiles (Fig. 5a). Notably, both profiles show a dynamic signal range exemplified by high-coverage regions sharply juxtaposed against largely unoccupied regions. At a large genomic window spanning multiple megabases, which represents numerous 500–50kb genomic bins input as features for dimensionality reduction, H3K27me3 occupies separate regions from H3K4me1–2-3. Distinct coverage patterns are maintained even at smaller regions occupied by both H3K27me3 and H3K4me1–2-3, such as directly over the *HOXA* genes, where H3K27me3 forms broader domains than H3K4me1–2-3. In genome-wide analysis, we found that H3K27me3 peaks were broader than H3K4me1–2-3 peaks (Fig. 5b), which likely facilitates SNP-based collision identification for H3K27me3, as broad peaks contain overlapping reads which cover more sequence space than narrow peaks. Accordingly, H3K27me3 peaks contained 45.75% of the 192,668 SNPs detected across the pooled chromatin profiles, whereas H3K4me1–2-3 peaks contained only 36.14% of SNPs. Raw reads were also enriched within their respective peak set (Fig. 5c), which suggested a general trend of mutual exclusivity across the two bulk profiles. Indeed, only 4117 peaks had at least 10% reciprocal intersection (Jaccard similarity = 0.06) between the 19,853 H3K27me3 peaks and 22,785 H3K4me1–2-3 peaks (Fig. 5d). Together, visual inspection of genomic coverage coupled with peak calling on the aggregated data demonstrates high signal-to-noise and confirms the specificity of these two antibody sets.

#### Cell-type annotation and confirmation with bulk projection

**Fig. 6:**
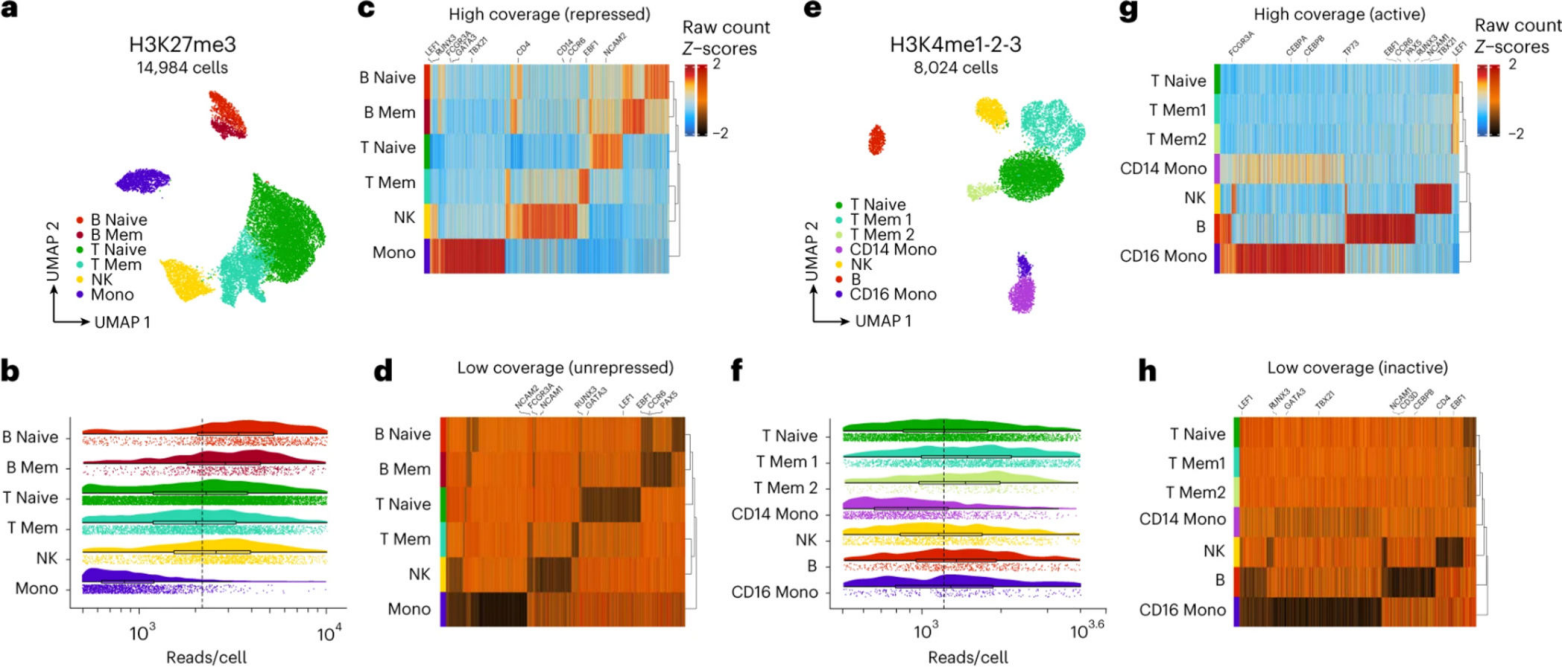
sciCUT&Tag supports graph-based clustering and cell type annotation of human PBMCs. a,UMAP embedding for H3K27me3 profiling in PBMCs colored by sciCUT&Tag cell type annotation based on gene coverage. Each point represents one cell. b,Reads/cell distribution per population in the H3K27me3 dataset. Black-dashed line is the dataset median. Density plot heights are normalized, box plots show the median with first and third quartiles. Each point represents one cell, c, Heat map of gene coverages per population for H3K27me3 with a log fold- change greater than 0.5 under a false discovery rate (FDR) of 0.01. d, Heat map of gene coverages per population for H3K27me3 with a log fold-change less than 0.5 under an FDR of 0.01. e, UMAP embedding for H3K4mel-2–3 profiling in PBMCs colored by sciCUT&Tag cell type annotation based on gene coverage. Each point represents one cell, f, Reads/cell distribution per population in the H3K4mel-2–3 dataset. Black dashed line is the dataset median. Density plot heights are normalized, box plots show the median with first and third quartiles. Each point represents one cell, g, Heat map of gene coverages per population for H3K4mel-2–3 with a log fold-change greater than 0.5 under a FDR of 0.01. h, Heat map of gene coverages per population for H3K4mel-2–3 with a log fold-change less than 0.5 under an FDR of 0.01. Heat map data are row-normalized, binary sorted by row and clustered by column. Mem, memory; NK, natural killer; Mono, monocyte.

Analysis of single-cell genomics data as described in step 148 generally includes cell-type annotation that is rooted in graph-based clustering. For graph-based clustering of sciCUT&Tag data, we used the approach implemented in Seurat^37^, and then compared common metrics across clusters, including information content (reads/cell) and gene coverage (Fig. 6). Computing coverage at marker genes this way provides a strong basis for cell type annotation because sciCUT&Tag maps the chemical impression of chromatin modifying enzymes within individual cells.

sciCUT&Tag profiling of H3K27me3 in PBMCs revealed six different cell populations with different similarities to one another and varying information content (Fig. 6a and b). We identified two B cell populations that clustered together and had higher reads/cell than the dataset median (Fig. 6b; black dashed line; median = 2116 reads/cell), as well as three more lymphoid populations with fewer reads/cell. We also identified one myeloid population with information content far below the median, which is consistent with previous H3K27me3 profiling of PBMCs^5^ and suggests that H3K27me3 may provide less signal in myeloid cells because they are less defined by the Polycomb group (PcG) chromatin modifiers that deposit H3K27me3^38^.

To annotate cell types in the H3K27me3 PBMC dataset, we computed gene coverage to identify genes with high and low differential coverage across clusters. For H3K27me3, high coverage indicates a ‘repressed’ status (Fig. 6c), whereas low coverage indicates the opposite (e.g., ‘unrepressed’; Fig. 6d). We found that differentially H3K27me3-enriched genes included common immune cell biomarkers (i.e., *LEF1, CD4, CD14, EBF1, NCAM2, etc.*).

We performed a similar analysis for H3K4me1–2-3 in PBMCs, which revealed seven different cell populations, some of which were not distinguished by H3K27me3. For example, H3K4me1–2-3 provided less granular resolution of B cells than H3K27me3, but more granular resolution of T cells, which separated into three distinct clusters (Fig. 6e) versus only two identified by H3K27me3. H3K4me1–2-3 also distinguished two myeloid populations, where H3K27me3 only distinguished one. Of note, the two myeloid populations identified by H3K4me1–2-3 exhibited different information content, with CD14 monocytes having lower reads/cell than CD16 monocytes. This suggests that H3K4 methylation distinguishes two myeloid populations by activity at only a few loci (Fig. 6f). Together, side-by-side analysis of H3K27me3 and H3K4me1–2-3 demonstrates that certain cell types are better distinguished by certain epitopes.

Importantly, gene coverage analysis validated our graph-based clustering by confirming that each cluster has a distinct coverage profile (Fig. 6c,d,g,h). Cell type annotation with H3K4me1–2-3 is straightforward because H3K4 methylation is mechanistically linked with chromatin remodeling^39^ and correlated with gene activation^40^. High H3K4me1–2-3 coverage can therefore be understood to mark ‘active’ genes (Fig. 6g), whereas low coverage means that a gene remains inactive (Fig. 6h). We applied the same list of gene biomarkers used for H3K27me3 to H3K4me1–2-3, which revealed a surprising overlap of crucial immune genes regulated by both PcG and Trithorax group (TrxG) chromatin modifiers^33^.

**Fig. 7:**
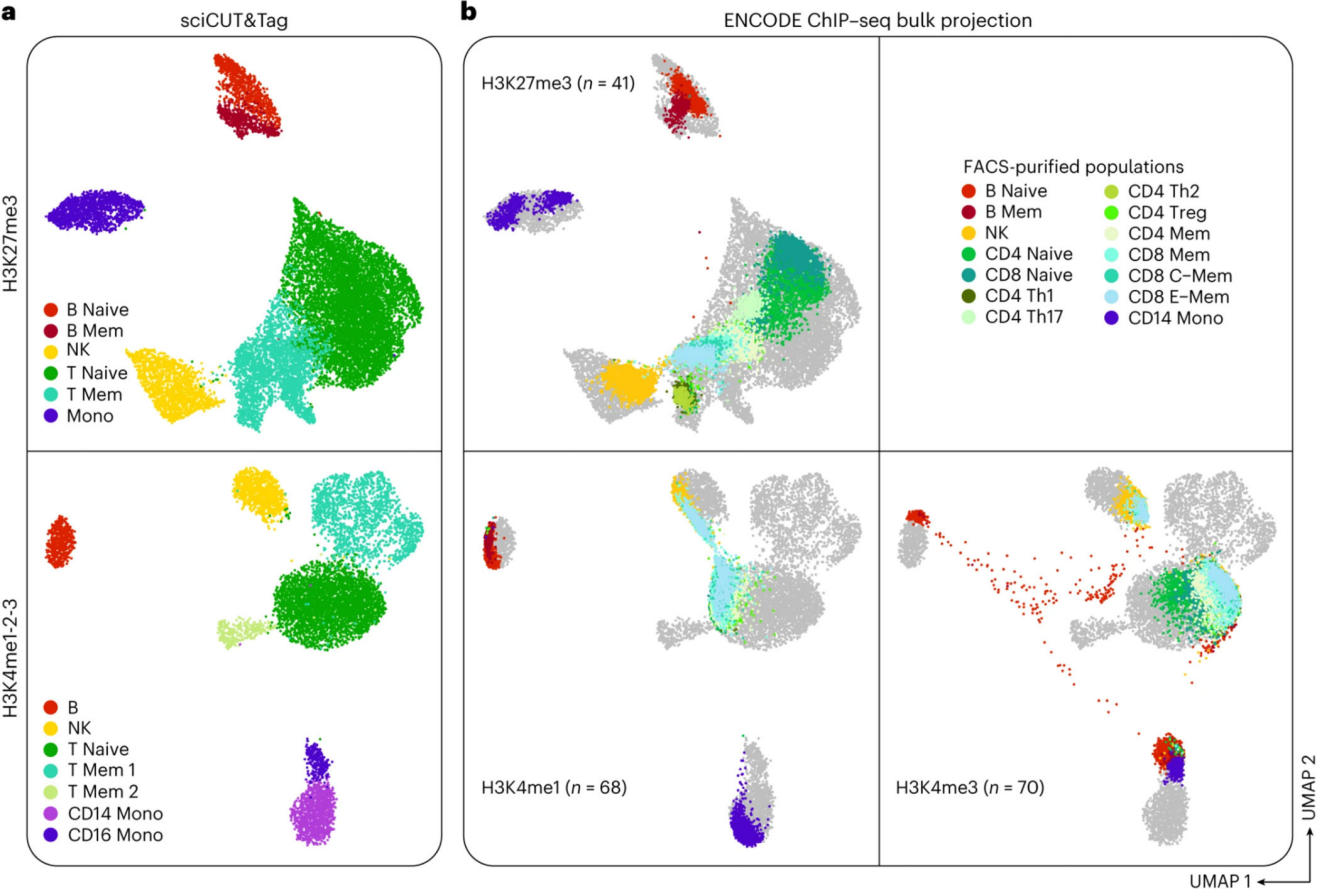
Orthogonal validation of sciCUT&Tag cell-type annotation by bulk projection of public data. a, UMAP embeddings for H3K27me3 (top) and H3K4mel-2–3 (bottom) profiling in PBMCs colored by cell type annotation based on gene coverage in sciCUT&Tag. Each point represents one cell, b, Bulk projections of ENCODE ChlP-seq into sciCUT&Tag UMAP embeddings. Counts per genomic bin (50 kb for H3K27me3,500 bp for H3K4mel and H3K4me3) were randomly split into 500 pseudo-cells per bulk dataset, then projected via sciCUT&Tag LSI representations. H3K27me3 profiles for FACS-purified populations were projected into H3K27me3 sciCUT&Tag (top) and H3K4mel (bottom left) and H3K4me3 (bottom right) profiles were projected into H3K4mel-2–3 sciCUT&Tag. Th, T helper; Treg, T regulatory; C-Mem, central memory; E-mem, effector memory.

We used bulk chromatin profiling data from the ENCODE project^8^ for orthogonal validation of CUT&Tag cell type annotation. Bulk chromatin profiling (*e.g.*, ChIP-seq, CUT&RUN, CUT&Tag) of any epitope can be randomly split into parts to simulate single-cell sparsity and projected into the same latent representation used for UMAP. To exemplify this validation technique, we projected ENCODE ChIP-seq data from immune populations purified by flow-assisted cell sorting (FACS) into our H3K27me3 and H3K4me1–2-3 UMAP embeddings (Fig. 7). For H3K27me3, this approach cleanly corroborated our cell type annotation based on gene coverages for all populations. For H3K4me1–2-3, however, bulk projection was less precise, as we profiled all forms of H3K4 methylation with sciCUT&Tag and only obtained bulk data for individual epitopes. Nonetheless, data for H3K4me1 and H3K4me3 projected into nearly all H3K4me1–2-3 sciCUT&Tag clusters and generally substantiated our gene coverage-based annotation.

In conclusion, the ability to obtain abundant and high-quality single-cell chromatin data at low cost with our sciCUT&Tag protocol enables the simultaneous profiling of multiple epitopes across multiple samples. Not only does combinatorial indexing on the ICELL8 make sciCUT&Tag tunable and transparent, but the easy-to-use pipeline for collision removal via *de novo* genotyping yields high-confidence data for tens of thousands of single cells per experiment.

## Supplementary Material

Supplementary Methods

Figures 1-7 (no captions)

Supplementary Table 1

Supplementary Methods and Figs. 1 and 2.

Reporting Summary

Supplementary Table 2

Supplementary Table 3

Supplementary Table 4

Supplementary Table 5

## Figures and Tables

**Table 2:** Troubleshooting table.

Steps	Problem	Possible reasons	Solution
15	An insufficient number of nuclei are recovered	The starting number of cells is too low for the nuclei-prep protocol.	Increase the number of cells used as input.
			Use a low input nuclei-prep method.
19	> 10% Nuclei remain in solution and are not bound by the beads	WGA-coated paramagnetic beads are suboptimal	Remake the WGA-coated beads
			Switch to ConA-coated MyOne-C1 beads
63	Very few tagmented nuclei pass through the 20 micron strainer	Nuclear lysis and aggregation	Limit work-times and harsh pipetting of the cells.
			Increase the cross-linking time from 2 to 3 min to prevent lysis
70	The number of nuclei is insufficient for 540 μL of 2.85 × 10^5^ nuclei / mL	Started protocol with fewer than 2 million nuclei, or nuclei were lost throughout the protocol.	Depending on the number of nuclei present, proceed with the whole sample in 540 μL.
76	Upon imaging, ICELL8 chip wells contain far more or less than 10 nuclei	There was an error in the quantification or dilution of tagmented nuclei prior to dispense	Redo the sample quantification and dilution, and dispense on a new ICELL8 chip.
126	The library yield is bellow 5 nM	Inefficient Tagmentation or Inefficient PCR.	Remake the sci-PA-Tn5 plate with a high efficiency prep of pA-Tn5
			Perform an SDS vs Triton-X titration to ensure concentrations are optimum for PCR.

## Data Availability

The primary sequencing data from the barnyard experiment, mixing human and mouse cell lines, as well as single donor and mixed donor PBMC sciCUT&Tag experiments have been deposited as paired-end fastq files that retain the unique cellular barcodes in the Gene Expression Omnibus accession GSE224579: https://www.ncbi.nlm.nih.gov/geo/query/acc.cgi?acc=GSE224579.

## Key references using this protocol

- Kaya-Okur HS, Wu SJ, Codomo CA, Pledger ES, Bryson TD, Henikoff JG, Ahmad K, Henikoff S. CUT&Tag for efficient epigenomic profiling of small samples and single cells. Nat Commun. 2019 Apr 29;10(1):1930. doi: 10.1038/s41467–019-09982–5. PMID: 31036827; PMCID: PMC6488672.
- Wu SJ, Furlan SN, Mihalas AB, Kaya-Okur HS, Feroze AH, Emerson SN, Zheng Y, Carson K, Cimino PJ, Keene CD, Sarthy JF, Gottardo R, Ahmad K, Henikoff S, Patel AP. Single-cell CUT&Tag analysis of chromatin modifications in differentiation and tumor progression. Nat Biotechnol. 2021 Jul;39(7):819–824. doi: 10.1038/s41587–021-00865-z. Epub 2021 Apr 12. PMID: 33846646; PMCID: PMC8277750.
- Meers MP, Llagas G, Janssens DH, Codomo CA, Henikoff S. Multifactorial profiling of epigenetic landscapes at single-cell resolution using MulTI-Tag. Nat Biotechnol. 2023 May;41(5):708–716. doi: 10.1038/s41587–022-01522–9. Epub 2022 Oct 31. PMID: 36316484; PMCID: PMC10188359.

## Key points

- This protocol describes a method for the single-cell profiling of epigenetic marks.
- Compared to other single-cell chromatin profiling methods, sciCUT&Tag can profile a greater number of cells while maintaining the optimum reaction chemistry for efficient PCR amplification of tagmented chromatin, thereby increasing the number of unique reads that are recovered per cell relative to droplet-based approaches.
