# Supplementary material for: Scalable single-cell profiling of chromatin modifications with sciCUT&Tag
Source: Nat Protoc. Author manuscript; Available in PMC 2024 Jul 8. (PMC11229882; doi:10.1038/s41596-023-00905-9)
Supplement: Supplementary Methods [file NIHMS1990485-supplement-Supplementary_Methods.docx]

### Supplementary Methods

**PBMC acquisition and processing:** Healthy adult consented donors at the Fred Hutchinson Cancer Center underwent venipuncture, and blood was collected using heparin-containing vacutainer tubes (Institutional Review Board IRB no. 0999.209). Mononuclear cells were harvested from peripheral blood using gradient centrifugation. Cells were then washed twice with PBS and lightly cross-linked nuclei were prepared as indicated in the protocol.

**Cell culture:** Human K562 cells were cultured according to the supplier’s protocol. H1 human embryonic stem cells were cultured in plates coated with Matrigel in mTeSR1 Basal Media containing mTeSR1 Supplement. The *KMT2A*r cell lines ML-2 and RS4;11 were obtained from the Bleakley laboratory at the Fred Hutchinson Cancer Research Center and were cultured in RPMI 1640 with glutamine and HEPES supplemented with 10% FBS. Mouse 3T3 cells were obtained from the Sarthy Lab at Seattle Children’s Hospital and were cultured in DMEM + Glutamax supplemented with 10% FBS and 1X Gibco Antibiotic-Antimyocotic. All cell lines were maintained in a cell Sanyo culture incubator with standard settings (37 °C with 5% CO_2_).
