# Supplementary material for: Scalable single-cell profiling of chromatin modifications with sciCUT&Tag
Source: Nat Protoc. Author manuscript; Available in PMC 2024 Jul 8. (PMC11229882; doi:10.1038/s41596-023-00905-9)
Supplement: Figures 1-7 (no captions) [file NIHMS1990485-supplement-Figures_1-7__no_captions_.pdf]

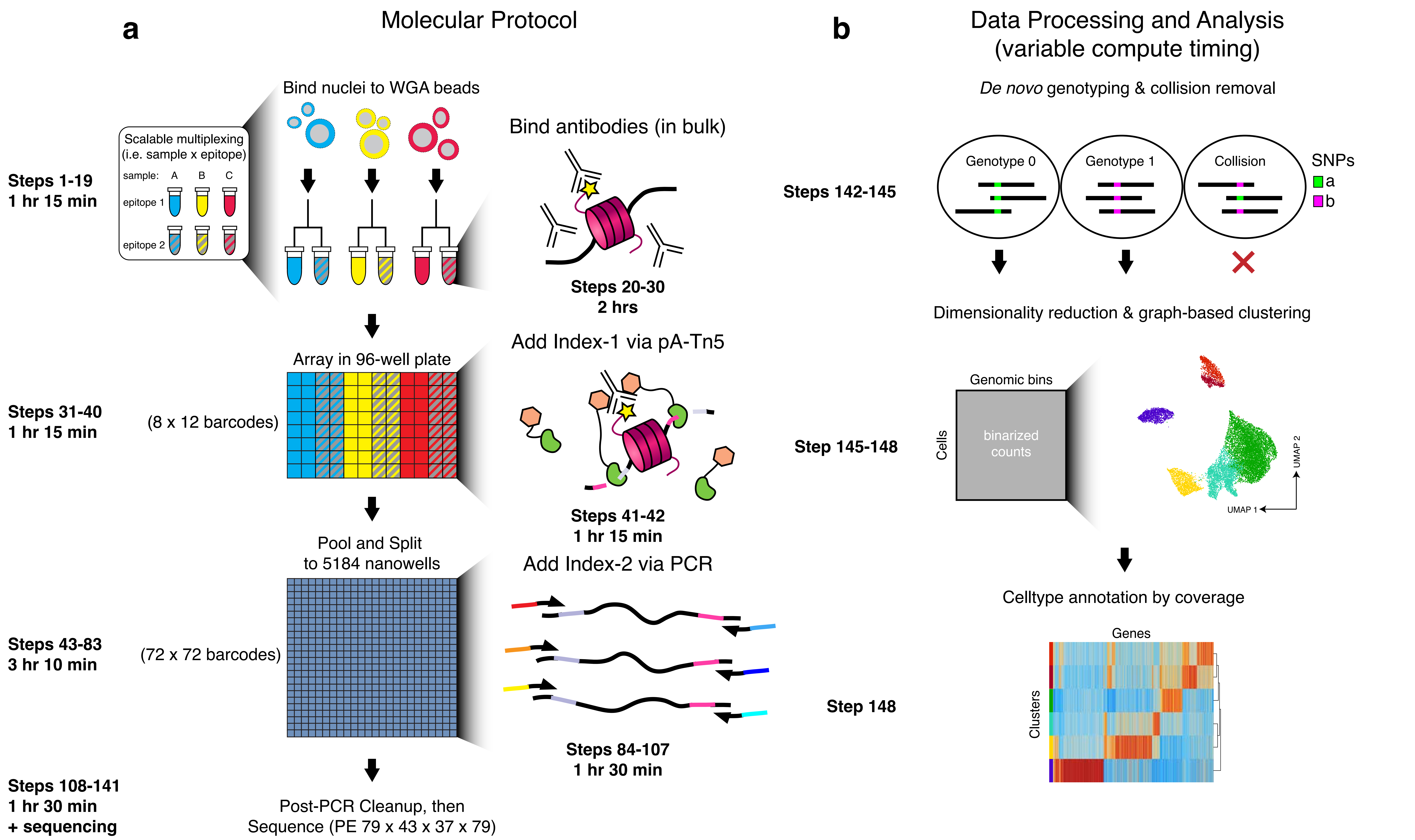
a
Molecular Protocol
Bind nuclei to WGA beads
Scalable multiplexing
(i.e. sample x epitope)
sample: A B C
epitope 1
epitope 2
Steps 1-19
1 hr 15 min
Bind antibodies (in bulk)
Steps 20-30
2 hrs
Array in 96-well plate
Steps 31-40
1 hr 15 min
(8 x 12 barcodes)
Add Index-1 via pA-Tn5
Steps 41-42
1 hr 15 min
Pool and Split
to 5184 nanowells
Steps 43-83
3 hr 10 min
(72 x 72 barcodes)
Add Index-2 via PCR
Steps 84-107
1 hr 30 min
Steps 108-141
1 hr 30 min
+ sequencing
Post-PCR Cleanup, then
Sequence (PE 79 x 43 x 37 x 79)
b
Data Processing and Analysis
(variable compute timing)
De novo genotyping & collision removal
Steps 142-145
Genotype 0
Genotype 1
Collision
SNPs
a
b
Dimensionality reduction & graph-based clustering
Step 145-148
Genomic bins
Cells
binarized
counts
UMAP 2
UMAP 1
Celltype annotation by coverage
Step 148
Genes
Clusters

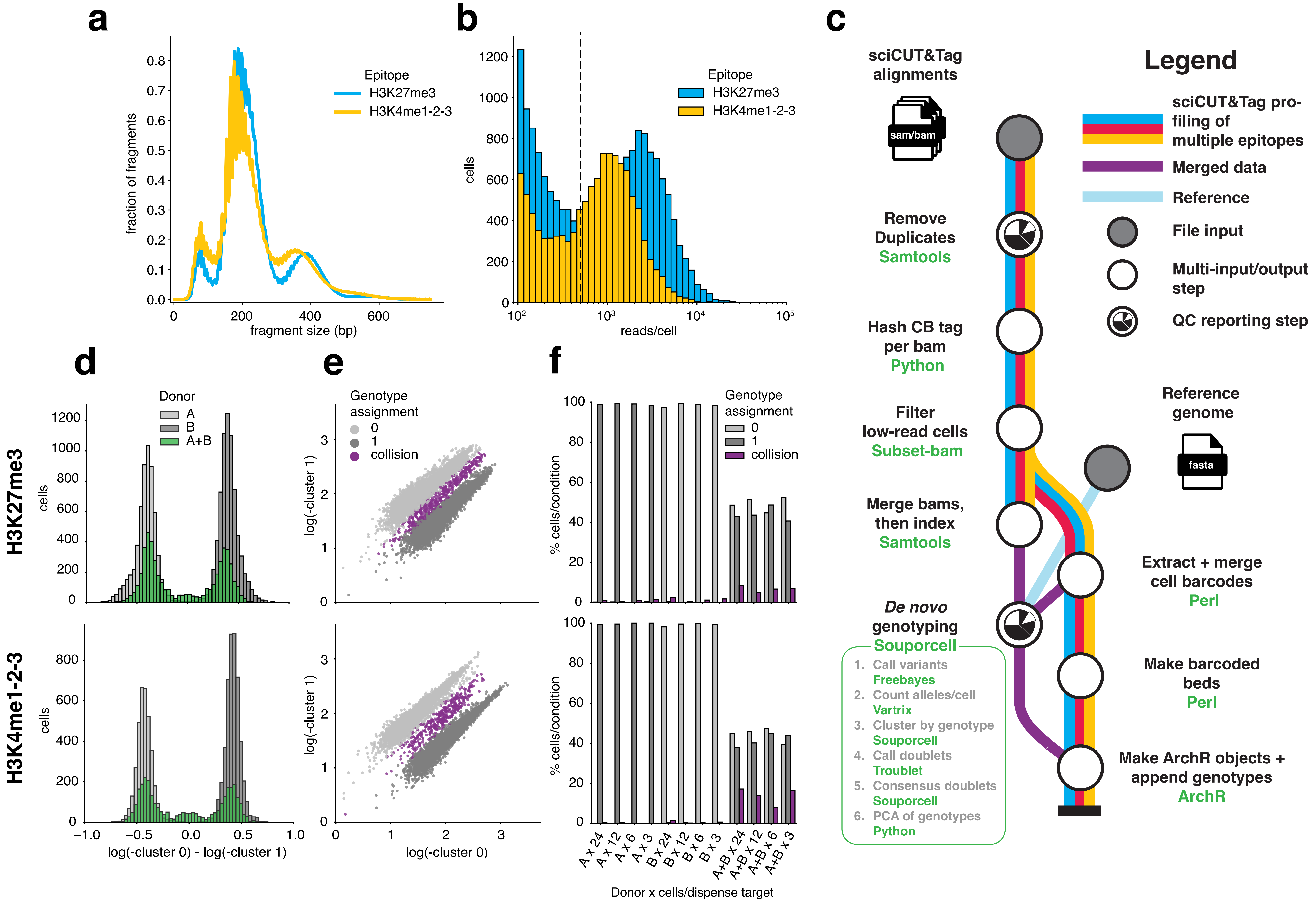

a
Epitope
H3K27me3
H3K4me1-2-3
fraction of fragments
fragment size (bp)
b
Epitope
H3K27me3
H3K4me1-2-3
cells
reads/cell
c
sciCUT&Tag alignments
sam/bam
Remove Duplicates
Samtools
Hash CB tag per bam
Python
Filter low-read cells
Subset-bam
Merge bams, then index
Samtools
De novo genotyping
Souporcell
1. Call variants
Freebayes
2. Count alleles/cell
Vartrix
3. Cluster by genotype
Souporcell
4. Call doublets
Troublet
5. Consensus doublets
Souporcell
6. PCA of genotypes
Python
Reference genome
fasta
Extract + merge cell barcodes
Perl
Make barcoded beds
Perl
Make ArchR objects + append genotypes
ArchR
Legend
sciCUT&Tag pro-filing of multiple epitopes
Merged data
Reference
File input
Multi-input/output step
QC reporting step
d
H3K27me3
H3K4me1-2-3
Donor
A
B
A+B
cells
log(-cluster 0) - log(-cluster 1)
e
Genotype assignment
0
1
collision
log(-cluster 1)
log(-cluster 0)
f
Genotype assignment
0
1
collision
% cells/condition
A x 24
A x 12
A x 6
A x 3
B x 24
B x 12
B x 6
B x 3
A+B x 24
A+B x 12
A+B x 6
A+B x 3
Donor x cells/dispense target

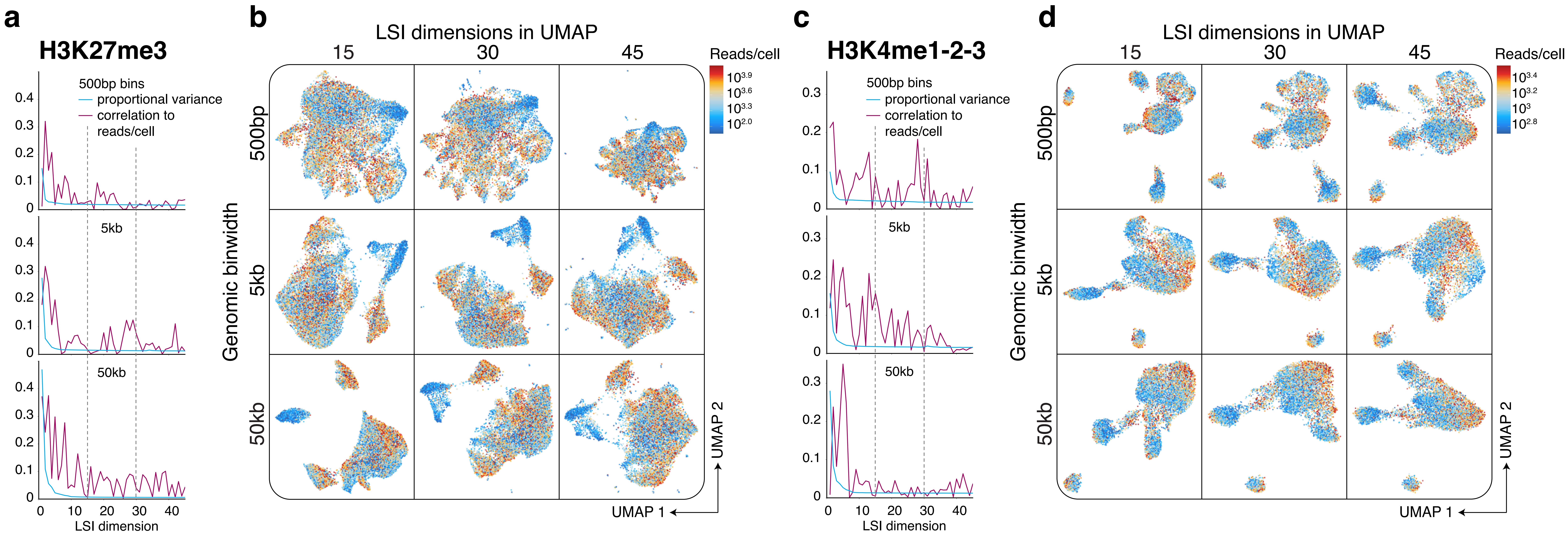

a
H3K27me3
500bp bins
proportional variance
correlation to reads/cell
5kb
50kb
LSI dimension
b
LSI dimensions in UMAP
15
30
45
Reads/cell
Genomic binwidth
500bp
5kb
50kb
UMAP 1
UMAP 2
c
H3K4me1-2-3
500bp bins
proportional variance
correlation to reads/cell
5kb
50kb
LSI dimension
d
LSI dimensions in UMAP
Reads/cell
Genomic binwidth
UMAP 1
UMAP 2

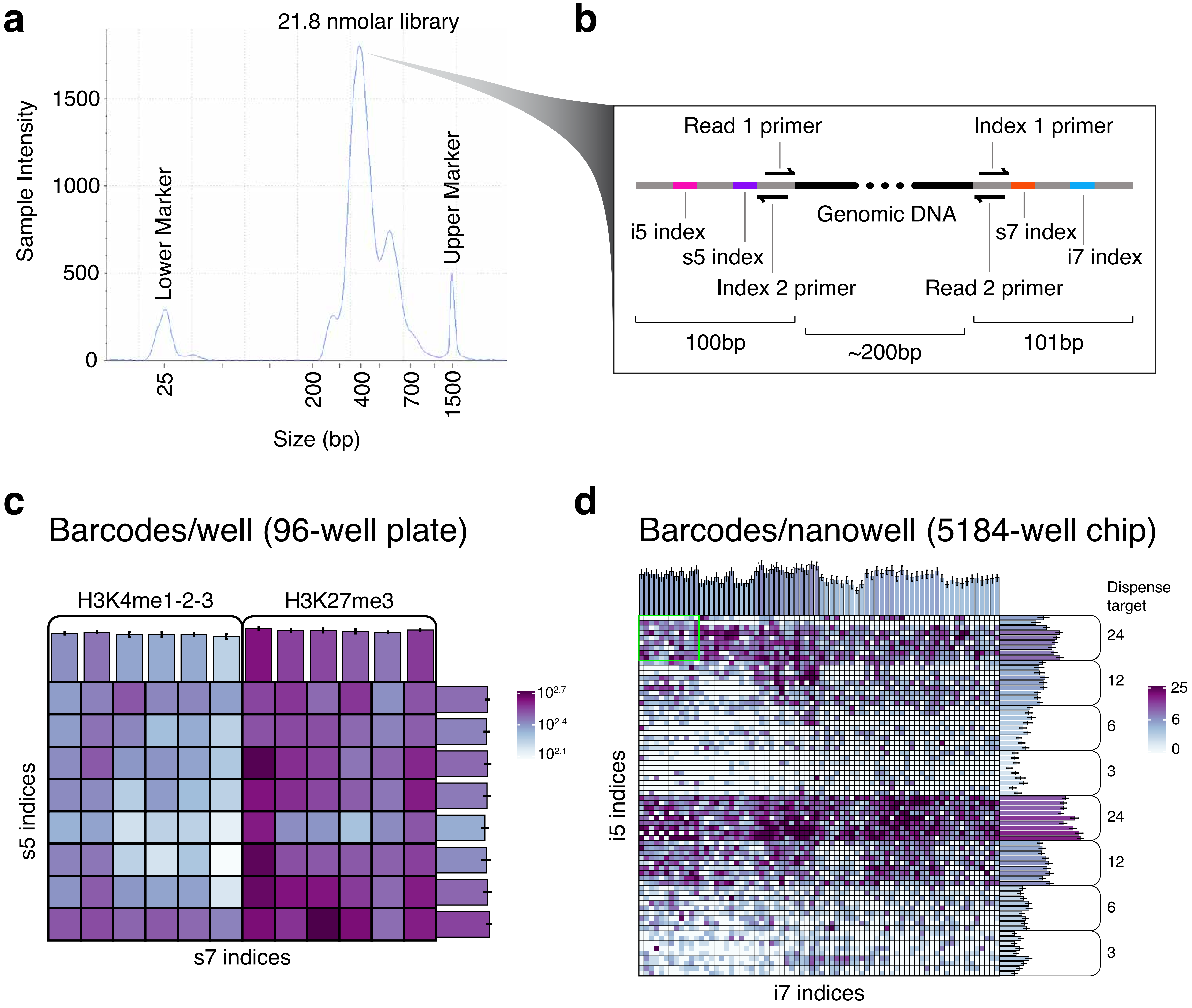
a
21.8 nmolar library
Sample Intensity
Lower Marker
Upper Marker
Size (bp)
b
Read 1 primer
Index 1 primer
Genomic DNA
i5 index
s5 index
Index 2 primer
Read 2 primer
s7 index
i7 index
100bp
~200bp
101bp
c
Barcodes/well (96-well plate)
H3K4me1-2-3
H3K27me3
s5 indices
s7 indices
d
Barcodes/nanowell (5184-well chip)
Dispense target
i5 indices
i7 indices

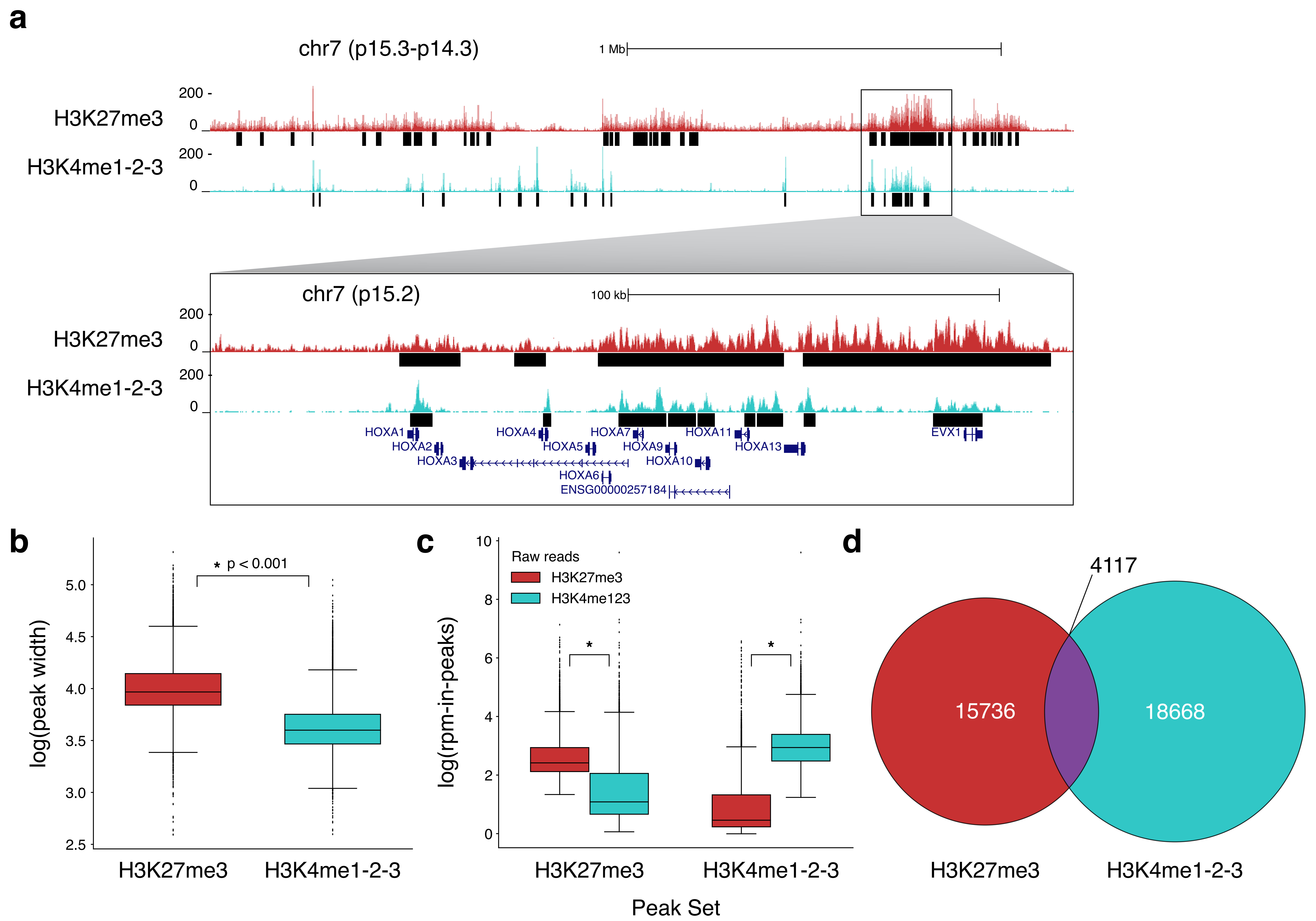

a
chr7 (p15.3-p14.3)
1 Mb
200 -
H3K27me3
0
200 -
H3K4me1-2-3
0
chr7 (p15.2)
100 kb
200
H3K27me3
0
200
H3K4me1-2-3
0
HOXA1
HOXA2
HOXA3
HOXA4
HOXA5
HOXA6
ENSG00000257184
HOXA7
HOXA9
HOXA10
HOXA11
HOXA13
EVX1
b
* p < 0.001
log(peak width)
5.0
4.5
4.0
3.5
3.0
2.5
H3K27me3
H3K4me1-2-3
c
Raw reads
H3K27me3
H3K4me123
log(rpm-in-peaks)
10
8
6
4
2
0
*
*
H3K27me3
H3K4me1-2-3
Peak Set
d
4117
15736
18668
H3K27me3
H3K4me1-2-3

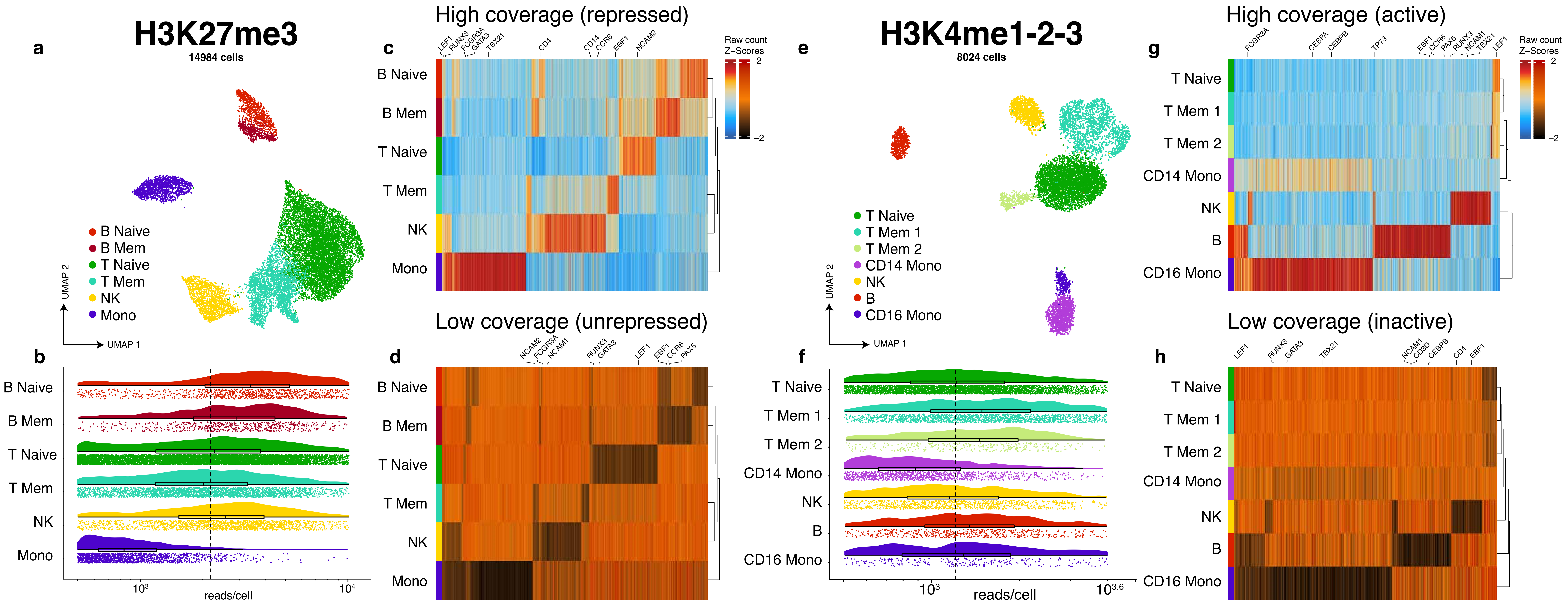

H3K27me3
14984 cells
a
B Naive
B Mem
T Naive
T Mem
NK
Mono
UMAP 1
UMAP 2
b
reads/cell
c
High coverage (repressed)
LEF1 RUNX3 FCGR3A GATA3 TBX21 CD4 CD14 CCR6 EBF1 NCAM2
Raw count Z-Scores
d
Low coverage (unrepressed)
NCAM2 FCGR3A NCAM1 RUNX3 GATA3 LEF1 EBF1 CCR6 PAX5
H3K4me1-2-3
8024 cells
e
T Naive
T Mem 1
T Mem 2
CD14 Mono
NK
B
CD16 Mono
f
reads/cell
g
High coverage (active)
FCGR3A CEBPA CEBPB TP73 EBF1 CCR6 PAX5 RUNX3 NCAM1 TBX21 LEF1
h
Low coverage (inactive)
LEF1 RUNX3 GATA3 TBX21 NCAM1 CD3D CEBPB CD4 EBF1

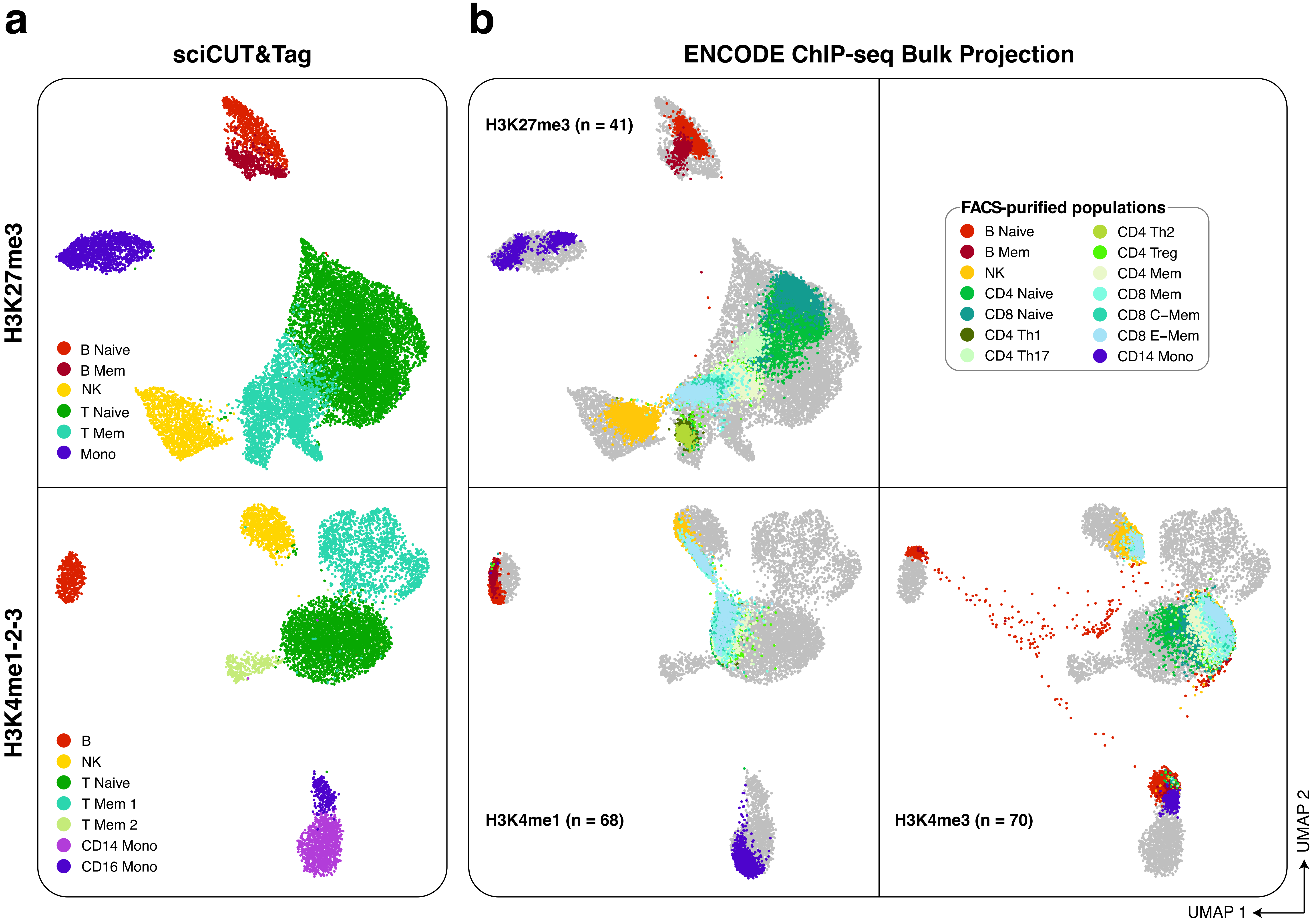

a
b
sciCUT&Tag
ENCODE ChIP-seq Bulk Projection
H3K27me3
H3K4me1-2-3
B Naive
B Mem
NK
T Naive
T Mem
Mono
B
NK
T Naive
T Mem 1
T Mem 2
CD14 Mono
CD16 Mono
H3K27me3 (n = 41)
H3K4me1 (n = 68)
H3K4me3 (n = 70)
FACS-purified populations
B Naive
B Mem
NK
CD4 Naive
CD8 Naive
CD4 Th1
CD4 Th17
CD4 Th2
CD4 Treg
CD4 Mem
CD8 Mem
CD8 C–Mem
CD8 E–Mem
CD14 Mono
UMAP 1
UMAP 2

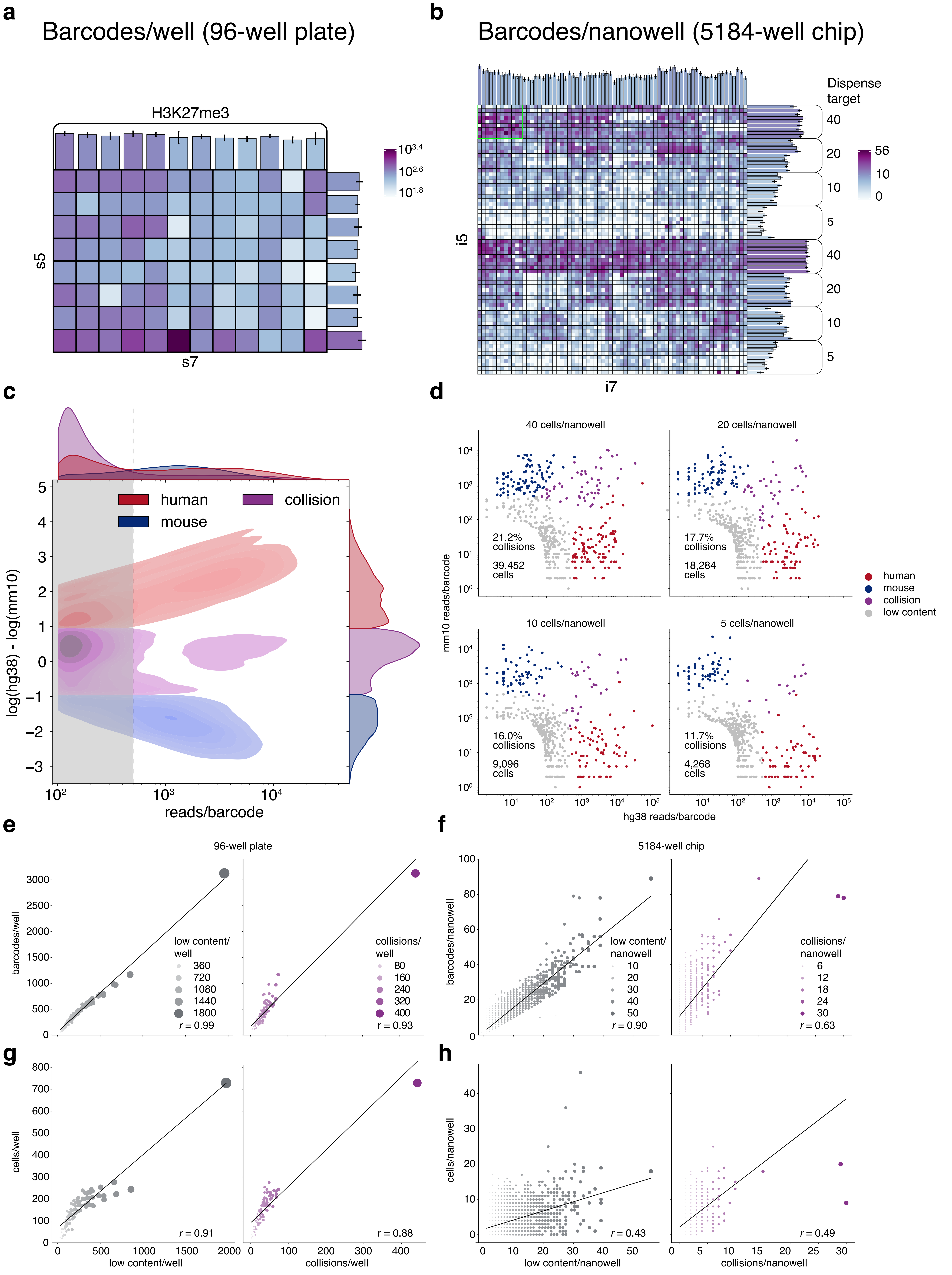
a
Barcodes/well (96-well plate)
H3K27me3
s5
s7
b
Barcodes/nanowell (5184-well chip)
Dispense target
40
20
10
5
40
20
10
5
56
10
0
i5
i7
c
human
collision
mouse
log(hg38) - log(mm10)
reads/barcode
d
40 cells/nanowell
20 cells/nanowell
10 cells/nanowell
5 cells/nanowell
21.2% collisions
39,452 cells
17.7% collisions
18,284 cells
16.0% collisions
9,096 cells
11.7% collisions
4,268 cells
mm10 reads/barcode
hg38 reads/barcode
human
mouse
collision
low content
e
96-well plate
barcodes/well
low content/well
360
720
1080
1440
1800
r = 0.99
collisions/well
80
160
240
320
400
r = 0.93
f
5184-well chip
barcodes/nanowell
low content/nanowell
10
20
30
40
50
r = 0.90
collisions/nanowell
6
12
18
24
30
r = 0.63
g
cells/well
r = 0.91
r = 0.88
low content/well
collisions/well
h
cells/nanowell
r = 0.43
r = 0.49
low content/nanowell
collisions/nanowell

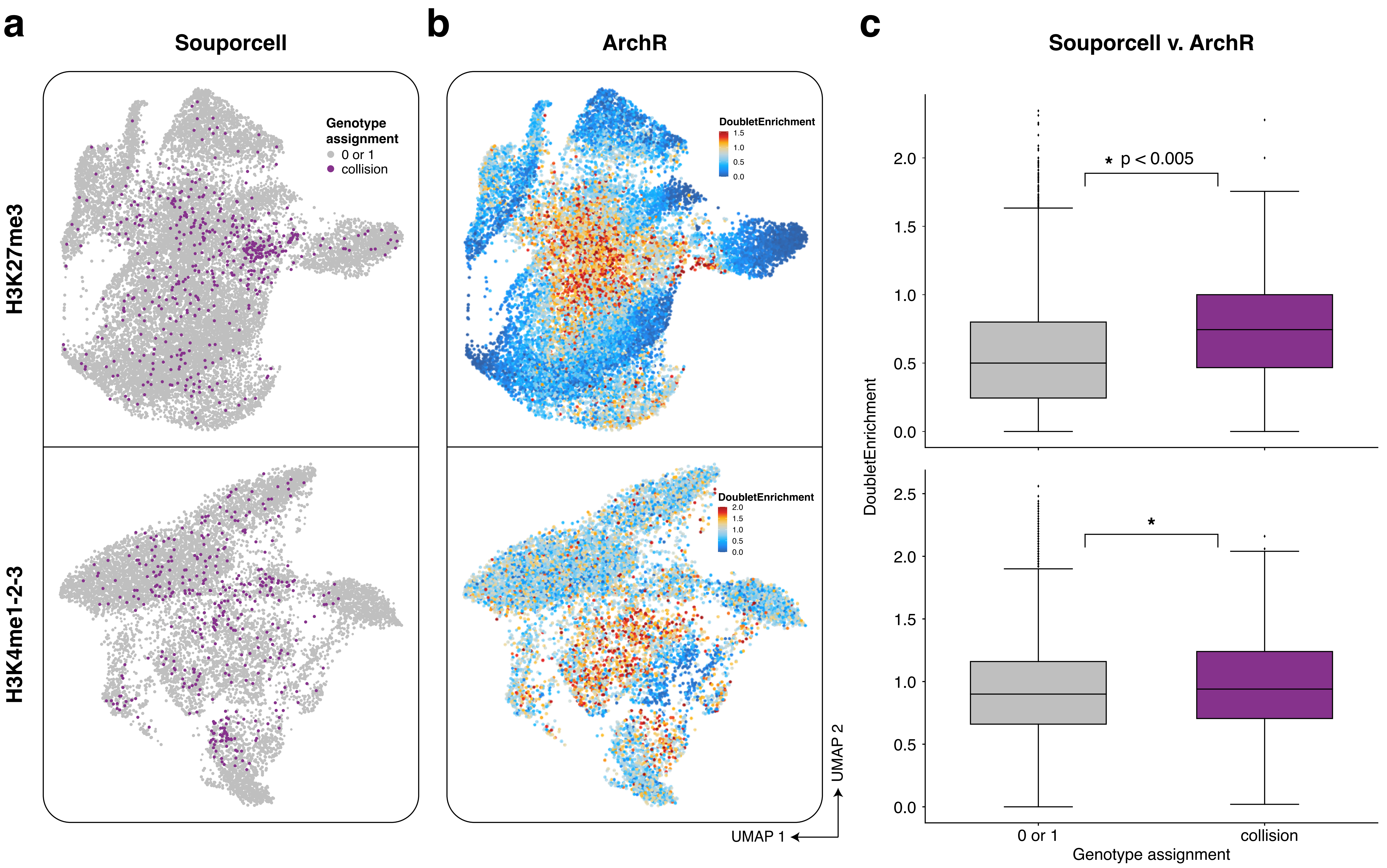
a
Souporcell
b
ArchR
c
Souporcell v. ArchR
Genotype assignment
0 or 1
collision
DoubletEnrichment
H3K27me3
H3K4me1-2-3
UMAP 1
UMAP 2
* p < 0.005
*
DoubletEnrichment
Genotype assignment
0 or 1
collision
