# Supplementary material for: Scalable single-cell profiling of chromatin modifications with sciCUT&Tag
Source: Nat Protoc. Author manuscript; Available in PMC 2024 Jul 8. (PMC11229882; doi:10.1038/s41596-023-00905-9)
Supplement: Supplementary Methods and Figs. 1 and 2. [file NIHMS1990485-supplement-Supplementary_Methods_and_Figs__1_and_2_.pdf]

nature protocols

https://doi.org/10.1038/s41596-023-00905-9

Protocol

Check for updates

Scalable single-cell profiling of chromatin modifications with sciCUT&Tag

In the format provided by the authors and unedited

Supplementary Methods

PBMC acquisition and processing: Healthy adult consented donors at the Fred Hutchinson Cancer Center underwent venipuncture, and blood was collected using heparin-containing vacutainer tubes (Institutional Review Board IRB no. 0999.209). Mononuclear cells were harvested from peripheral blood using gradient centrifugation. Cells were then washed twice with PBS and lightly cross-linked nuclei were prepared as indicated in the protocol.

Cell culture: Human K562 cells were cultured according to the supplier's protocol. H1 human embryonic stem cells were cultured in plates coated with Matrigel in mTeSR1 Basal Media containing mTeSR1 Supplement. The *KMT2A*r cell lines ML-2 and RS4;11 were obtained from the Bleakley laboratory at the Fred Hutchinson Cancer Research Center and were cultured in RPMI 1640 with glutamine and HEPES supplemented with 10% FBS. Mouse 3T3 cells were obtained from the Sarthy Lab at Seattle Children's Hospital and were cultured in DMEM + Glutamax supplemented with 10% FBS and 1X Gibco Antibiotic-Antimyocotic. All cell lines were maintained in a cell Sanyo culture incubator with standard settings (37 °C with 5% CO_2).

Extended Data

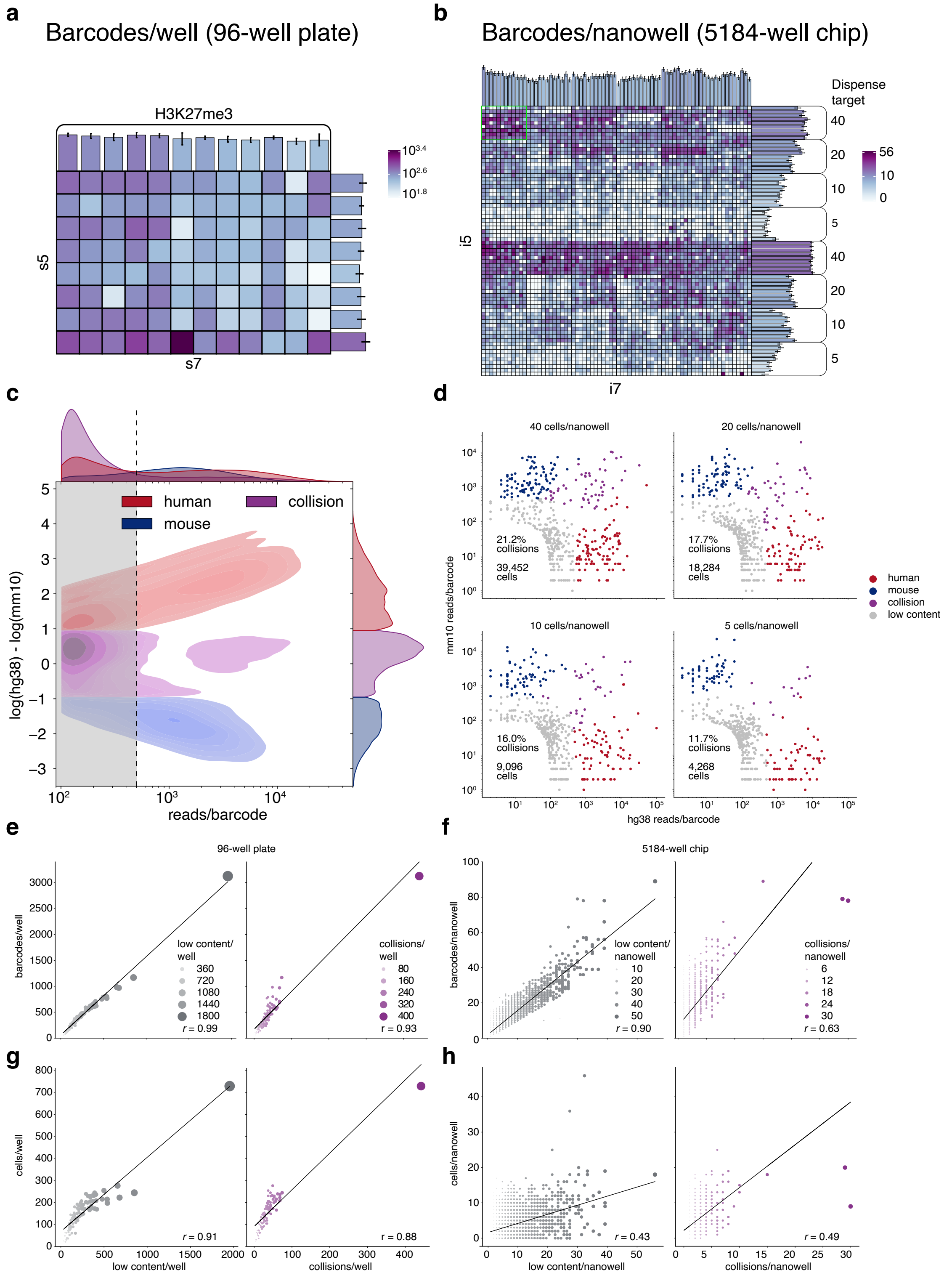

Supplementary Figure 1 | Mapping the determinants of collisions and low-content barcodes in sciCUT&Tag a) 96-well plate heatmap shaded per well by unique barcode combinations with greater than 100 reads recovered by sequencing in the barnyard experiment that mixed human and mouse cell lines at a 1:1 ratio. Marginal bar plots show the mean number of unique combinations recovered per s5 and s7 index and are shaded accordingly. Marginal bar plots for the s7 indices are labeled by profiled epitope. Error bars are standard deviation. **b)** 5184-well chip shaded by unique barcode combinations with greater than 100 reads recovered by sequencing per well in the barnyard experiment. Marginal bar plots show the mean number of unique combinations recovered per column and row and are shaded accordingly. Marginal bar plots along the y-axis are labeled by the cell/well dispense target. Error bars are standard error. **c)** Bivariate density plot of the log differential of reads/barcode that uniquely align to hg38 or mm10 versus total reads/barcode. Barcodes with >90% of reads uniquely aligned to hg38 are called as human (red), likewise for mm10 (mouse, blue), and barcodes with <90% of reads uniquely aligned to either hg38 or mm10 are called as a collision (purple). Marginal plots show univariate density. A black dashed line marks 500 reads/barcode and barcodes with less than 500 reads are shaded gray. **d)** Scatter plots of 500 randomly sampled barcodes for each dispense target. Each dot represents one unique barcode combination plotted according to number of reads that uniquely map to hg38 or mm10. Barcodes with >90% of reads uniquely aligned to hg38 are called as human (red), likewise for mm10 (mouse, blue), and otherwise are called as a collision (purple). Low-content barcodes, which are excluded from downstream analysis, are colored gray. Rate of collision is percentage of collisions per total cells. Cells/chip is estimated as four times the yield from a quarter of one 5184-well chip. **e)** Barcodes/well linearly correlated against low-content barcodes/well (gray) and collisions/well (purple) for the 96-well plate. Each dot represents one well of the 96-well plate and is sized and shaded according to low-content barcodes/well and collisions/well, respectively. **f**) same as **c** for the 5184-well chip. **e)** High-content (e.g., >500 reads/barcode) cells/well linearly correlated against low-content barcodes/well (gray) and collisions/well (purple) for the 96-well plate. Each dot represents one well of the 96-well plate and is sized and shaded according to low-content barcodes/well and collisions/well, respectively. **f**) same as **c** for the 5184-well chip. Correlations are Pearson's R and the best-fit linear regression is shown in black for **e-h**.

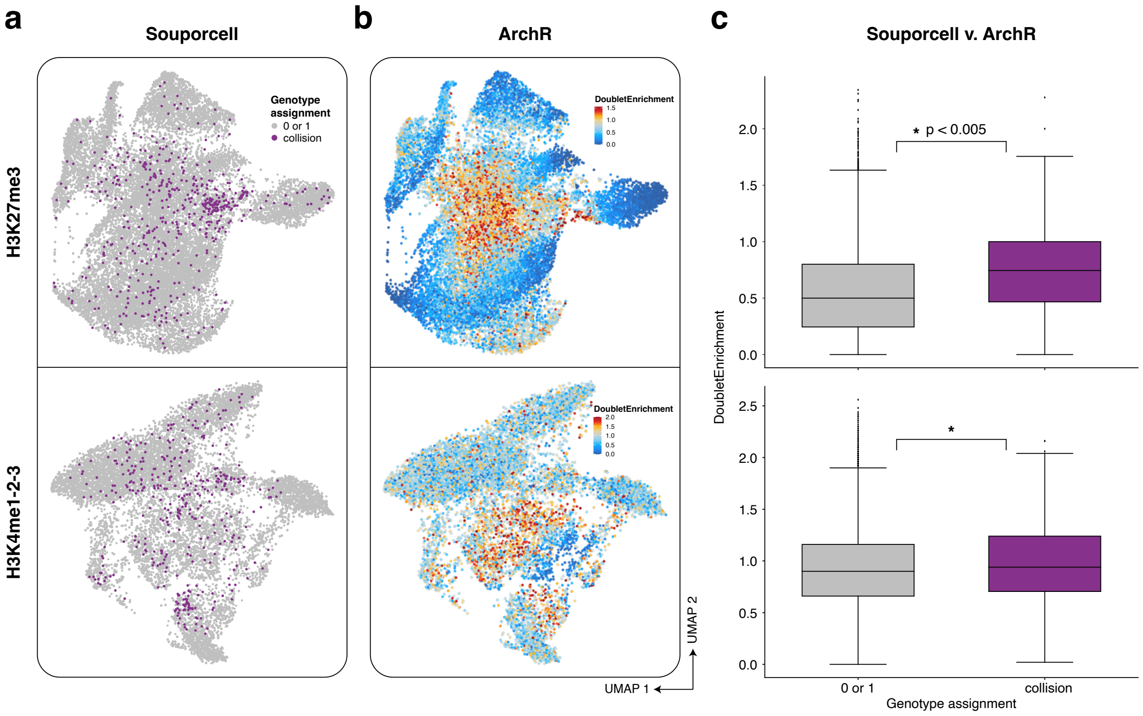

Supplementary Figure 2 | Souporcell identifies collisions also detected by artificial nearest neighbor testing in ArchR a) UMAPs for H3K27me3 (top) and H3K4me1-2-3 (bottom) profiling in PBMCs that include the collisions identified by Souporcell[22] (purple). **b)** Same as **a** but UMAPs are colored by DoubletEnrichment score from addDoubletScores() in ArchR[25]. **c)** Comparison of the ArchR DoubletEnrichment score in Souporcell collisions v. non-collisions (0 or 1 genotype assignment).
